# Nanoarchitectonics: Complexes and Conjugates of Platinum Drugs with Silicon Containing Nanocarriers. An Overview

**DOI:** 10.3390/ijms22179264

**Published:** 2021-08-26

**Authors:** Kinga Piorecka, Jan Kurjata, Wlodzimierz A. Stanczyk

**Affiliations:** Centre of Molecular and Macromolecular Studies, Polish Academy of Sciences, Sienkiewicza 112, 90-363 Lodz, Poland; jkurjata@cbmm.lodz.pl (J.K.); was@cbmm.lodz.pl (W.A.S.)

**Keywords:** anticancer platinum drugs, nanoconjugates, nanocomplexes synthesis and properties, silicon containing nanocarriers

## Abstract

The development in the area of novel anticancer prodrugs (conjugates and complexes) has attracted growing attention from many research groups. The dangerous side effects of currently used anticancer drugs, including cisplatin and other platinum based drugs, as well their systemic toxicity is a driving force for intensive search and presents a safer way in delivery platform of active molecules. Silicon based nanocarriers play an important role in achieving the goal of synthesis of the more effective prodrugs. It is worth to underline that silicon based platform including silica and silsesquioxane nanocarriers offers higher stability, biocompatibility of such the materials and pro-longed release of active platinum drugs. Silicon nanomaterials themselves are well-known for improving drug delivery, being themselves non-toxic, and versatile, and tailored surface chemistry. This review summarizes the current state-of-the-art within constructs of silicon-containing nano-carriers conjugated and complexed with platinum based drugs. Contrary to a number of other reviews, it stresses the role of nano-chemistry as a primary tool in the development of novel prodrugs.

## 1. Introduction

Chemotherapy is the most important treatment of the most cancer cases, but still, the search for the improvement of anticancer drugs and prodrugs is an important challenge for contemporary science [1]. Cisplatin (Peyrone’s chloride) and other platinum drugs belong to a group of widely-used chemotherapeutics. Although, their use in conventional therapy has several limitations as the safety window is narrow due to a number of dangerous side effects exerted by these drugs, including nephrotoxicity, myelosuppression, peripheral neuropathy, ototoxicity and nausea, as well as lack of selectivity [2,3,4]. Therefore, currently, we are witnessing a rapid development of drug delivery systems, i.e., drug conjugates and non-covalent complexes with nanoparticles (NPs) (nanocarriers), e.g., such as gold, mesoporous silica nanoparticles, and silsesquioxanes, polymers, and dendrimers [5,6]. The advantage of using nanoparticles is the selective drug delivery and its controlled release in pathological cells or tissues. Nanochemistry [7] nanotechnology [8] and nanomedicine [9,10] offer nowadays a variety of systems that are intensively studied as delivery vehicles, modulating systemic cytotoxicity and mediating sustained drug release in tumor tissues. They provide new opportunities for avoiding drawbacks of conventional therapies using innovative solutions for selective and side effects free therapy of cancer. Therefore, the development of novel nano-systems and their biologic evaluation is an area of great importance for contemporary society.

**Scheme 1:**
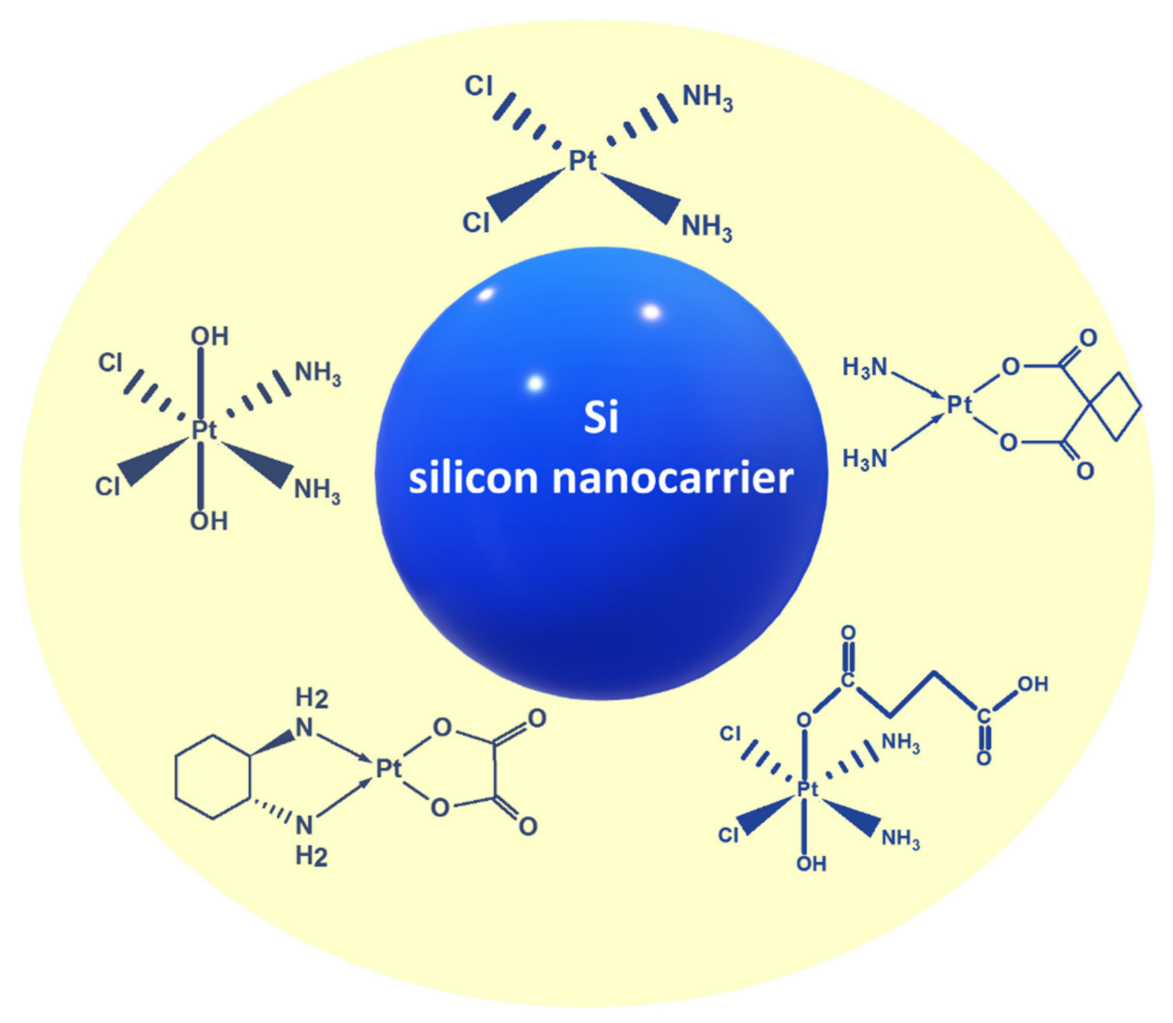
Illustration of silicon-containing constructs nanocarriers conjugated and complexed with platinum-based drugs.

In this review, we report on the state of the art in the area of silicon containing nanocarriers for platinum drugs delivery (Scheme 1). Over the past decades, silica nano-complexes and nanonjugates with drugs have attracted growing attention among scientists. They are considered to be materials that can meet the challenges of modern medicine in the fight against cancer. Mesoporous silica nanoparticles, due to such features as biocompatibility, non-toxicity, chemical stability, adjustable pore sizes, large pores volume, large surface area and functionalization, are used for design of drug delivery systems in biomedical applications [11,12,13,14,15,16,17]. Basically, inorganic systems have several important physical and chemical advantages. Both modified mesoporous silica and polyhedral silsesquioxanes (POSS) present good in vivo and in vitro compatibility. In vitro and in vivo studies suggest that, e.g., cisplatin-delivering polysilsesquioxane particles offer important improvements compared to standard chemoradiation therapy with cisplatin [5,18], and are nowadays regarded as the next generation of nanomaterials for biomedical applications [19]. They can be easily functionalized with fluorescent or tumor-targeting moieties, selectively interacting with and overexpressing tumor cell receptors [20,21]. Therefore, both these types of “silicon” species can be used as effective drug carriers for platinum-based anticancer drugs. Additionally they can be modified with polyethylene glycol and targeting agents to improve solubility, circulation in blood flow, as well as allow active, and not only passive or triggered delivery [18,19,22,23,24]. They are also biodegradable and break down into non-toxic orthosilicic acid that is easily excreted [25]. It should be underlined that recent review papers scarcely describe a synthetic approach to this important group of platinum drugs conjugates and complexes, and their application in biological research.

## 2. Platinum Drugs Conjugates

**Figure 1:**
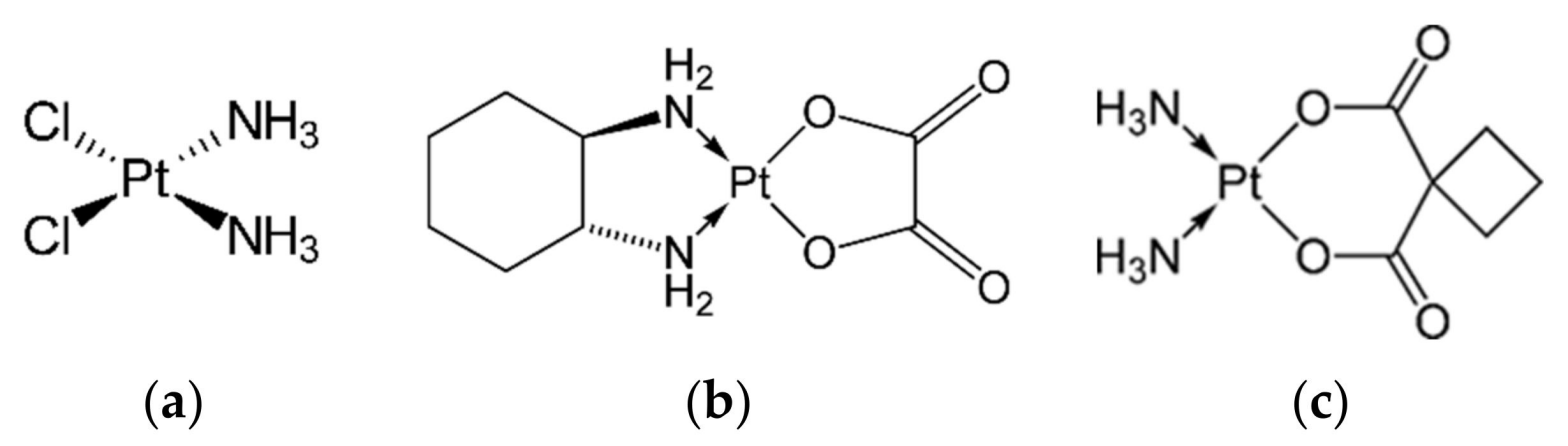
Platinum(II) anticancer drugs (**a**) cislatin, (**b**) oxaliplatin, (**c**) carboplatin.

In order to achieve conjugation with nanocarriers the structure of crucial clinical platinum drugs such as cisplatin (a) oxaliplatin, and (b) carboplatin (c) (Figure 1) has to be modified with suitable functionalized linkers [26,27].

This concept is based on covalent binding of the drug to relevant nanocarrier. Modification results in formation of a variety of Pt(IV) prodrugs that on their own [2,28,29], even before attachment to carriers, might improve pharmacological activity compared to platinum (II) systems used currently. They show kinetic inertness that makes them more stable in blood circulation and are activated via intracellular reduction to square planar platinum(II) compounds after cellular uptake [30,31,32,33].

### 2.1. Silica-Pt(IV) Conjugates

**Figure 2:**
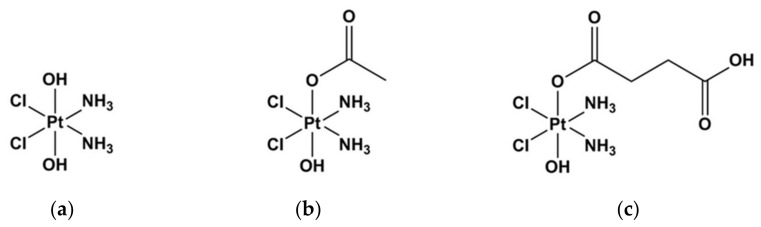
Basic platinum(IV) synthons applied in preparation of nanoconjugates (**a**–**c**).

Several platinum(IV) medicinal bioinorganic synthons were currently applied in formation of covalent bonds with a number of nanocarriers involving silica. The basic ones were oxoplatin [34,35] (a) hydroxyl(acetoxy)cisplatin [36] (b) and succinic anhydride-functionalized hydroxylcisplatin [37] (c) (Figure 2).

**Figure 3:**
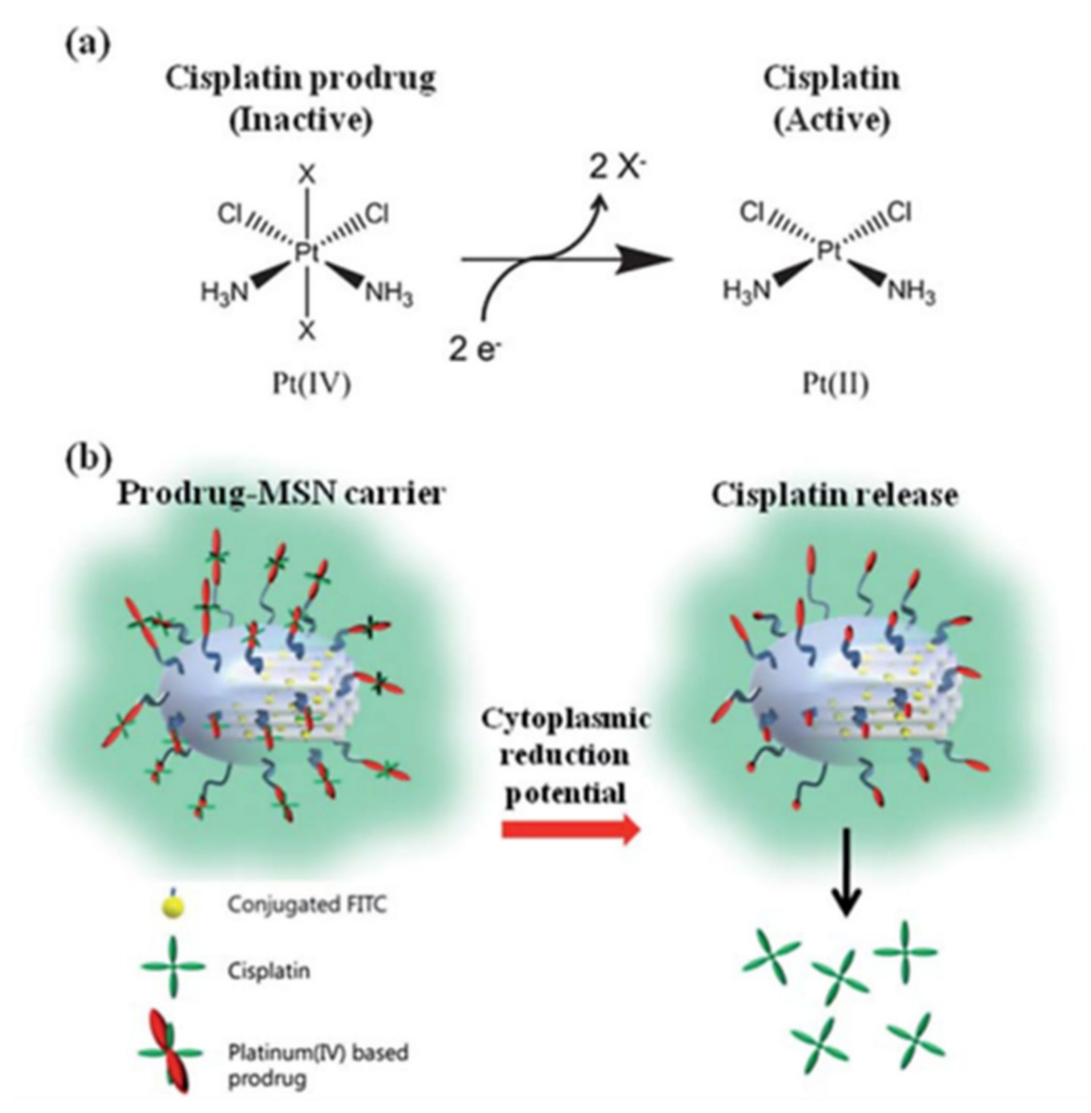
Pt(IV)-based prodrug consisting of square-planar cisplatin and two axial ligands, X. The two electron reduction to square-planar Pt(II), with loss of the axial ligands, is also shown (**a**). Scheme of the reduction potential-responsive cisplatin release from a prodrug-MSN carrier is presented, FITC -fluorescein isothiocyanate (**b**) [38].

The use of a Pt(IV)-based prodrugs that involves oxidation of cisplatin has gained growing attention. The higher the oxidation state of Pt in prodrugs leads to two additional coordination sites that gives the freedom to introduce various functionalities as required to overcome the solubility, easy and effective conjugation of prodrugs to delivery nanocarriers and targeting agents. Upon cellular internalization the prodrug can be transformed back into the active reduced form of cisplatin as effectuated by the stimuli-responsive trigger under reductive cellular environment [38] (Figure 3).

Mesoporous Silica Nanoparticles (MSN) are suggested as excellent candidates for the construction of efficient multifunctional platforms serving delivery of drugs and diagnostic nanoprobes [39]. MSN contain a high number of Si-OH surface groups providing attachment sites for different types of functionalization and the drug carriers have drawn considerable attention in cisplatin delivery, and its controlled release [38,40]. Fluorescein isothiocyanate (FITC) modified MSN (F-MSN) were synthesized according to a standard method [41]. In the first step fluorescein-modified 3-aminopropyltriethoxysilane (APTES) was prepared by mixing and stirring FITC with APTES. Next, the modified silane and tetraethoxysilane (TEOS) were added to an aqueous solution of n-cetyammonium bromide (CTAB) and sodium hydroxide. The surface of the obtained F-MSN was modified with amine functions by subsequent refluxing of F-MSN with APTES to yield nanoparticles with aminopropyl modified surface (F-MSN-AP). The amount of amine groups bound to the surface was determined by fluorescamine assay. In the final step, the conjugation of such the surface modified F-MSN-AP with [Pt(NH_3_)_2_Cl_2_(O_2_CH_2_CH_2_CH_2_CO_2_H)_2_] was achieved in a typical amide coupling reaction. Cell toxicity tests have shown that the prodrug-conjugated nanoparticles (F-MSN-prodrug) exhibited 63 times lower IC_50_ value compared to cisplatin in HeLa, 5.7 times in A549 and 14.5 times in MCF-7 cell lines. Moreover, the location of F-MSN-prodrug system can be easily monitored [38].

**Figure 4:**
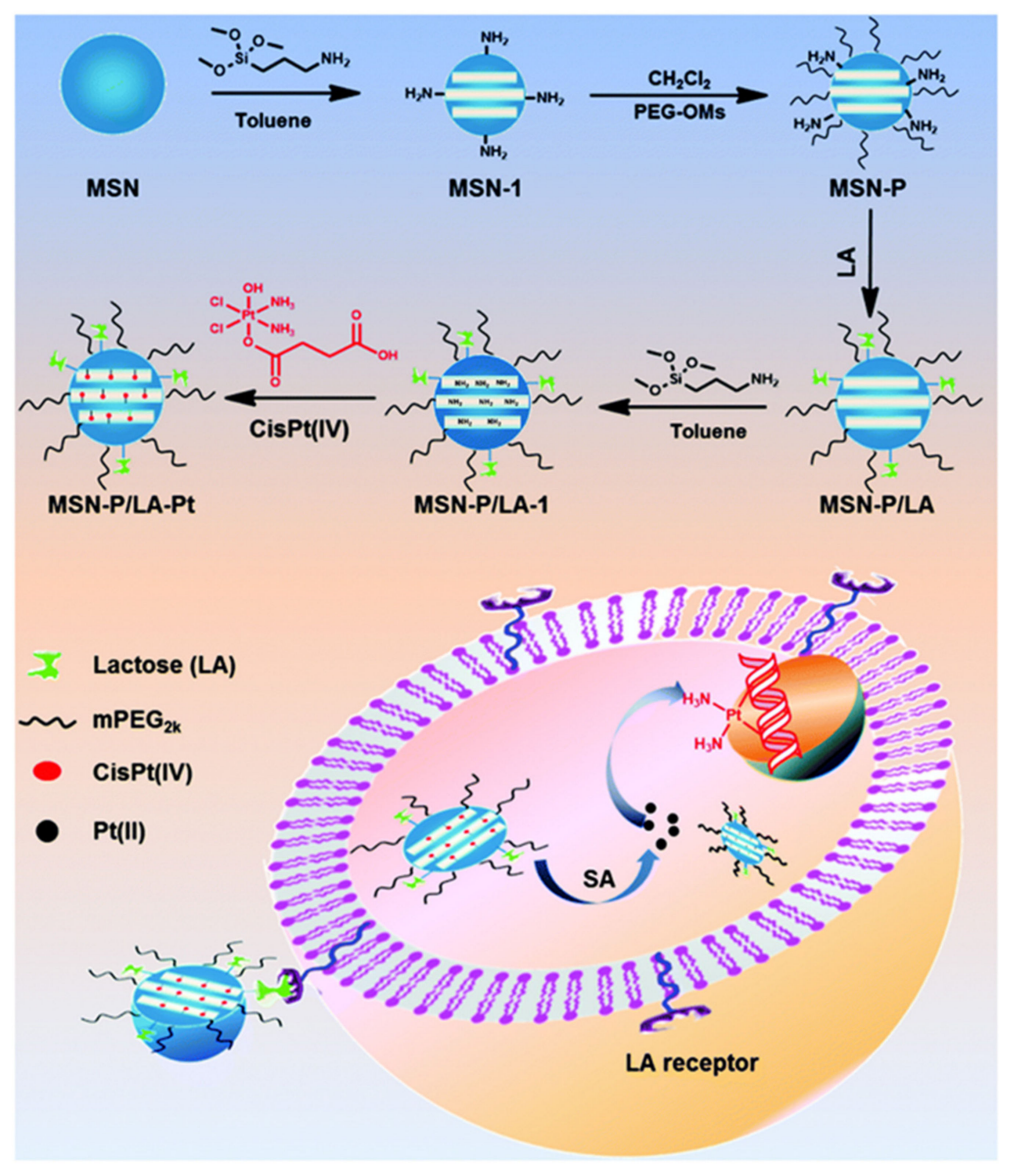
Tailor-made synthesis of mesoporous silica nanoparticles for platinum(IV) prodrug delivery and glutathione or sodium ascorbate (SA) induced reduction process to Pt(II) in tumor environment [22].

The multifunctional character of recent nano-systems is well illustrated by synthesis of targeted mesoporous silica nanoparticles with lactose mediated effect that were studied in delivery of platinum(IV) prodrug for liver anticancer therapy [22,37] (Figure 4). The authors presented a multi-step modification of standard MSN made from TEOS, conjugating Pt(IV) prodrug—[Pt(NH_3_)_2_Cl_2_(OH)(O_2_CH_2_CH_2_CH_2_CO_2_H)] inside nanoparticles and targeting agent—lactose (LA) with MSN surface. Additionally, the solubility of the nano-system and circulation time in blood was improved via covalent attachment of poly(ethylene glycol) (mPEG2k). The subsequent preparation steps of the MSN-P/LA-Pt particles are shown in Figure 4. The MTT assay against HepG-2 cancer cells revealed that the IC_50_ value of the synthesized Pt(IV) containing nanoparticles was 10.46 µM and was slightly higher than that of cisplatin. The authors claim that the advantage of the conjugate is the enhanced Pt accumulation in tumor site, as shown by in vivo studies when compared to cisplatin, and additionally, much lower systemic toxicity.

**Figure 5:**
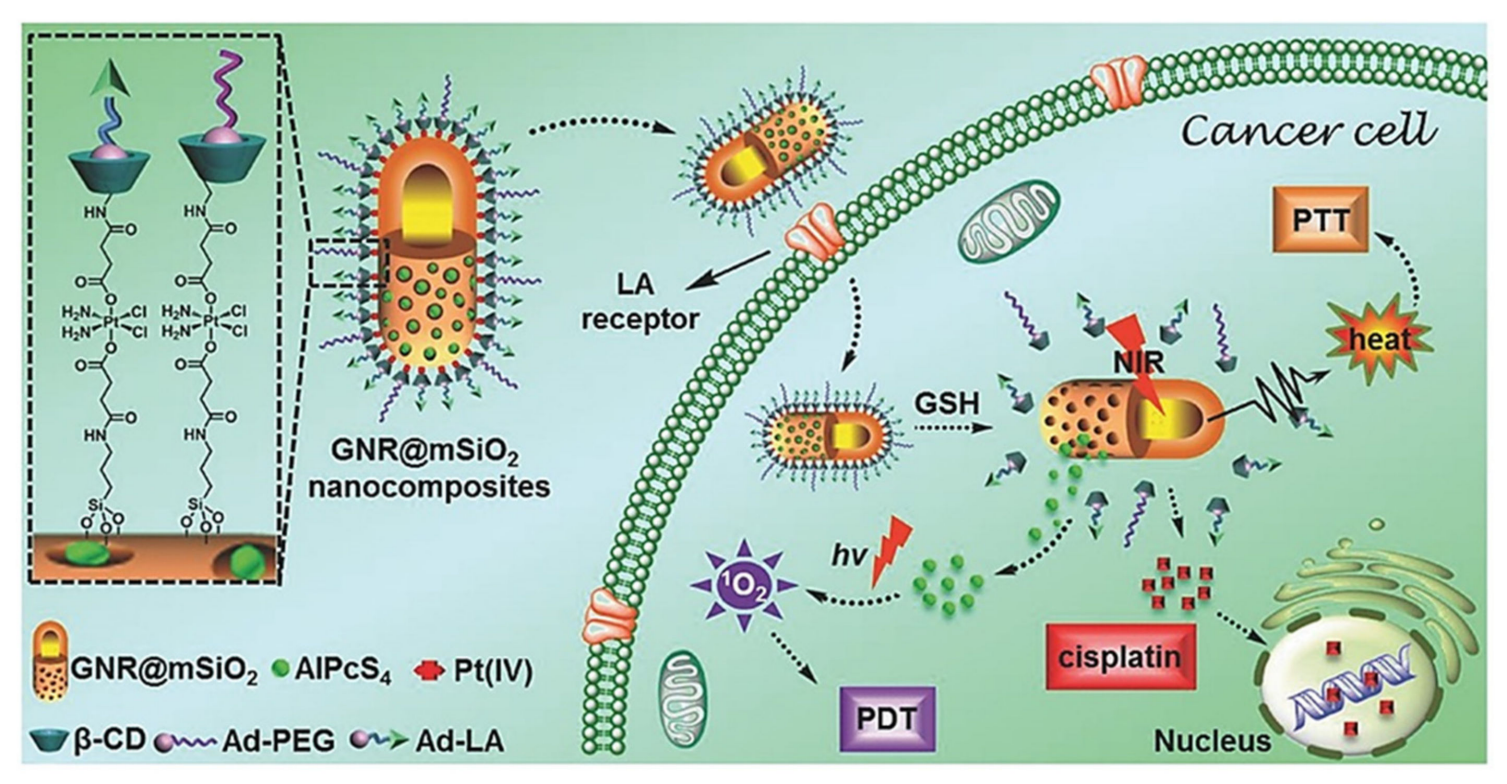
Schematic illustration of platinum nanoconjugate for tri-model high-performance tumour therapy [42].

A multifunctional nanocomposite based on mesoporous silica covered gold nanorod system (MSGNR), effective in oncotherapy, has been developed. It combines photothermal, photodynamic and chemotherapeutic treatment approach targeting specifically hepatoma cells. MSGNR nanocarrier was synthesized from HAuCl_4_ in CTAB solution by reduction with NaBH_4_. The obtained GNR were coated with SiO_2_ using TEOS/MeOH solution in the presence of NaOH. The surface of silica covered gold nanorods was then modified with APTES and coupled with [Pt(NH_3_)_2_CL_2_(O_2_CH_2_CH_2_CH_2_CO_2_H)_2_] to give MSGNR-Pt(IV)-COOH and subsequently mono-6-ethylenediamine-β-cyclodextrin was grafted as a “gatekeeping” moiety to avoid premature release of photosensitizer (PS). The nano-system was also equipped with a (PS)—[Al(III) phthalocyanine chloride tetrasulfonic acid, AlPcS_4_] entrapped in the porous structure of silica coating. β-Cyclodextrin was decorated with adamantane conjugated poly(ethylene glycol) (Ad-PEG) and lactobionic acid (Ad-LA) via formation of host-guest complexes to allow, respectively, long term circulation and tumor targeting. The structure of nano-system and its performance model are presented in Figure 5 [42].

The in vitro activity of the conjugate, determined by MTT assay revealed that at the Pt concentration of 2.85 µg/mL, without irradiation, the HepG2 cells survival was ~70%. However, once dual laser illumination (at 808 nm and 660 nm) was applied, the cell killing reached 92%. It points to high effectiveness of triple combination therapy as heat and ROS generation enhanced destruction of cells. The in vivo experiments also revealed improved accumulation of the nano-system in tumor, superior tumor growth inhibition and reduced systemic toxicity.

**Figure 6:**
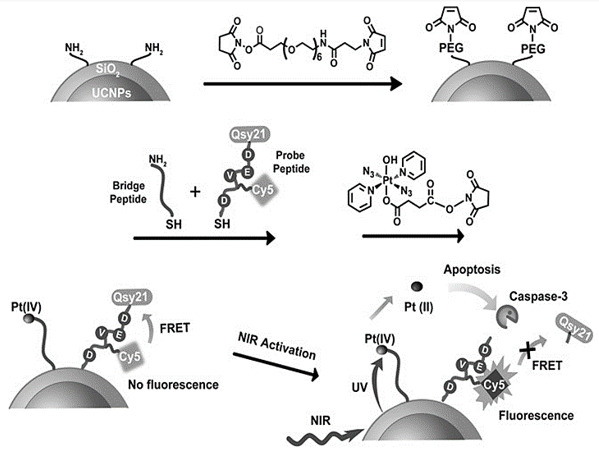
Scheme of NIR activation of platinum (IV) prodrug grafted on up conversion luminescent nanoparticles (UCNPs@SiO_2_) [44].

Up conversion nanoparticles (UCNPs) have become in the past years the promising vehicles of diverse drug molecules [9], including the remote control of platinum (IV) prodrugs activation. Lanthanide-doped (yttrium, ytterbium, thulium) luminescent UCNPs absorb NIR light and convert it into broad range emissions [43]. NIR triggered selective activation of Pt(IV) prodrug grafted on core-shell silica coated UCNPs into Pt(II) drug leads to cellular apoptosis at target tumor sites (A2780 cells). The system also allows for non-invasive optical imaging of apoptosis using caspase-targeted activity-based probes (Figure 6) [44]

The silica coated UCNPs@SiO_2_ bearing amino moieties was obtained by reverse micelles method with TEOS and APTES [45]. These were functionalized with a bridge peptide sequence (KKKKKC) through an oligo(ethyl glycol) (dPEG6) linker that contained a maleimide group to react with respective thiols (Figure 6). Then the antitumor prodrug, a photoactivatable Pt (IV) complex trans,trans,trans-[Pt(N_3_)_2_(OH)(O_2_CCH_2_CH_2_CO_2_H)(py)_2_] with one carboxyl group at axial position modified with N-hydroxysuccinimide (NHS) was conjugated with UCNPs@SiO_2_ surface.

### 2.2. Silica-Pt(II) Conjugates

**Figure 7:**
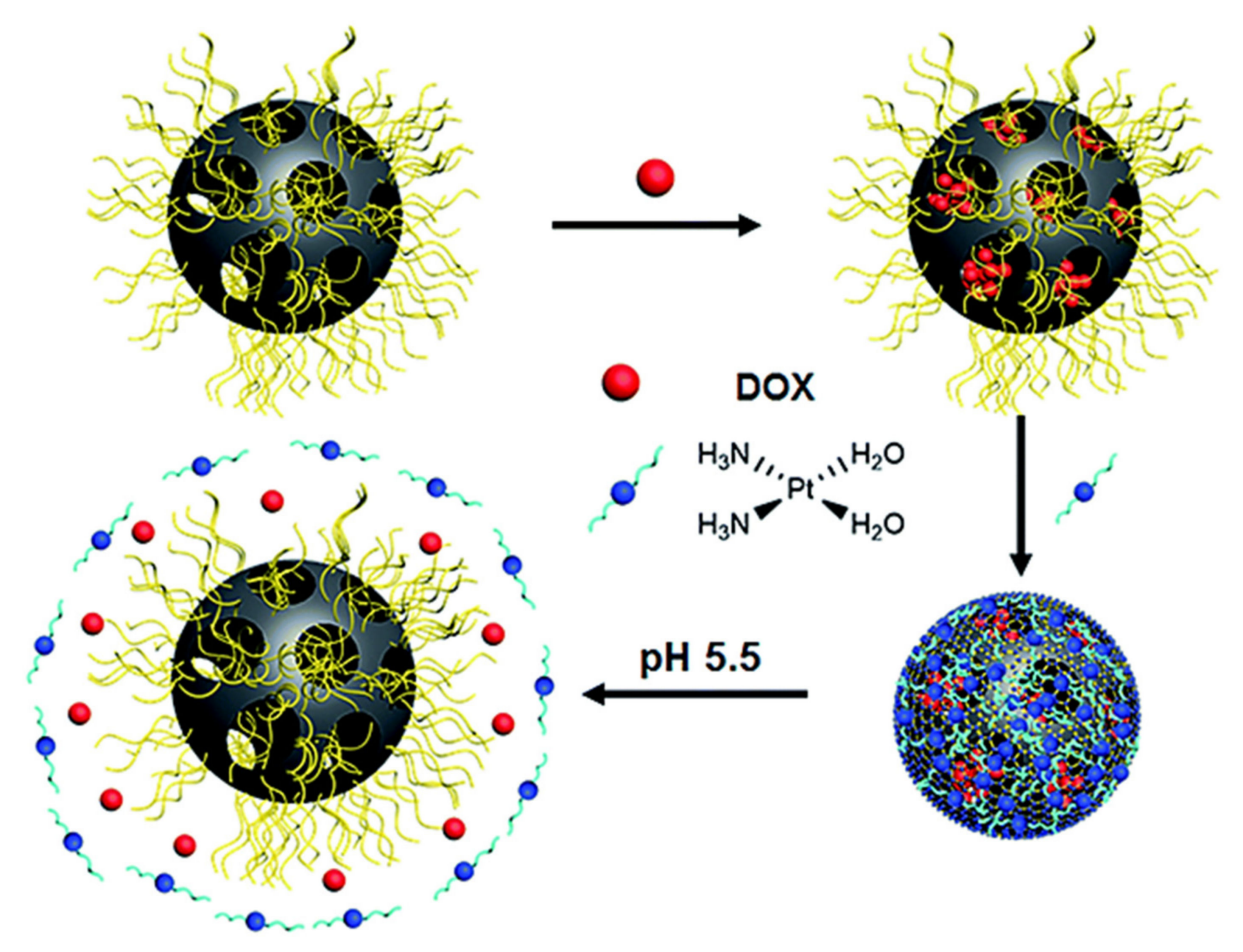
Schematic illustration of the synthesis and drug release behaviour of Pt@PAA-MSNDOX [46].

A novel multicomponent therapeutic platform was designed for combined delivery of doxorubicin (DOX) and cisplatin based on poly(acrylic acid) modified MSN (Pt@PAA-MSNDOX) (Figure 7).

**Figure 8:**
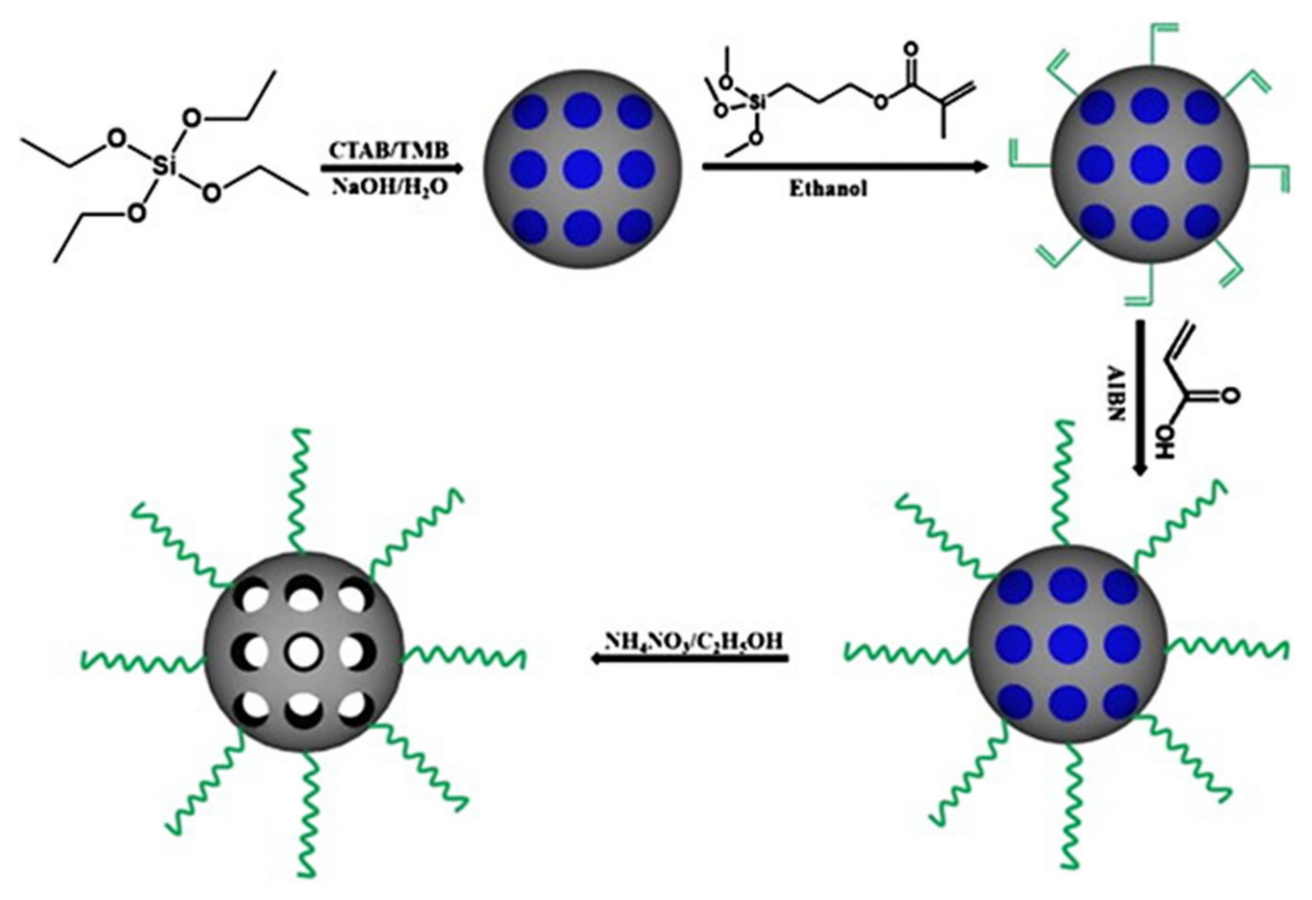
Preparation of nanoparticles with SiO_2_ core and poly(acrylic acid) shell (PAA-MSN) [46,47,48].

The synthesis of mesoporous silica nanoparticle core with crosslinked poly(acrylic acid) shell has been earlier described by Yang’s group [46,47] (Figure 7). The core was made from TEOS using CTAB and trimethylbenzene (TMB) as templates. The resultant nanoparticles underwent modification of surface with methacryloxypropyltrimethoxysilane (MPS) by post-grafting method, yielding double bond functionalized nanoparticles (MSN-MPS). They were subsequently used for distillation-precipitation polymerisation of acrylic acid (AA) in the presence of azobisisobutyronitrile (AIBN) as an initiator and N,N’-methylenebisacrylamide (MBA) as crosslinker. In a final step the templates were removed by refluxing in a solution of ammonium nitrate in ethanol (Figure 8).

**Figure 9:**
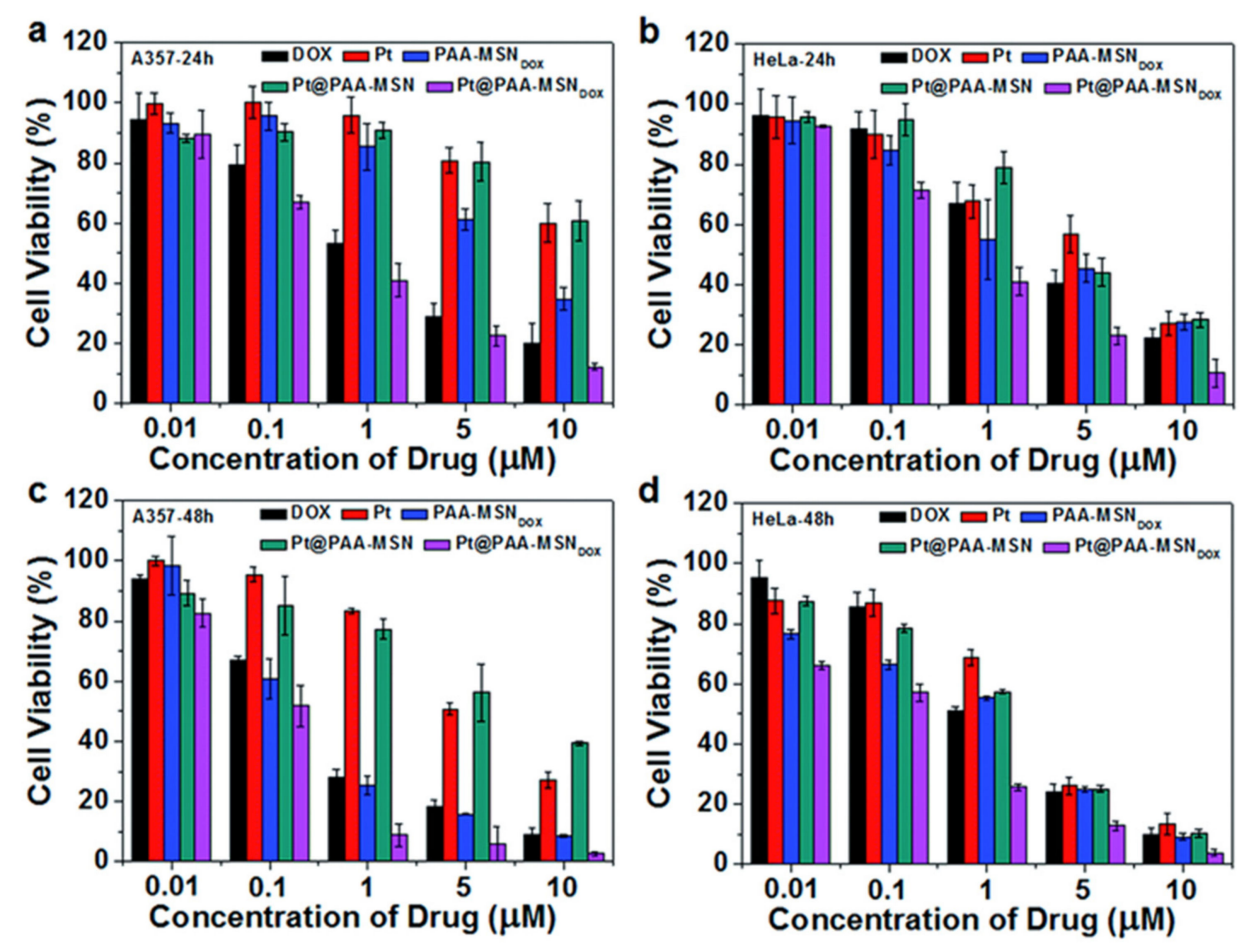
In vitro viability studies of A357 and HeLa cells against free single drugs, Pt@PAA-MSN, PAA-MSNDOX and Pt@PAA-MSNDOX: (**a**) A357 cells incubated for 24 h; (**b**) HeLa cells incubated for 24 h; (**c**) A357 cells incubated for 48 h; (**d**) HeLa cells incubated for 48 h [46].

DOX was encapsulated in the MSN core mesopores while cisplatin was reacted with aqueous silver nitrate to form cis-diamminediaqua platinum(II) that was later conjugated with carboxyl groups on the surface of PAA-MSN (Figure 7). The extensive in vitro cytotoxicity CCK-8 assays against A357 and Hela cells were performed, not only for Pt@PAA-MSNDOX and free drugs. They also involved single drug-loaded nanoparticles—PAA-MSNDOX and Pt@PAA-MSN (Figure 9).

Single-loaded nanoparticles and free drugs exhibited similar cytotoxicity, which points to an efficient drugs release, while Pt@PAA-MSNDOX system showed the highest activity in both cell lines. It appears that this novel dual-loaded silica nanocarrier presents a great potential as a pH responsive delivery system for treatment of cancer.

**Figure 10:**
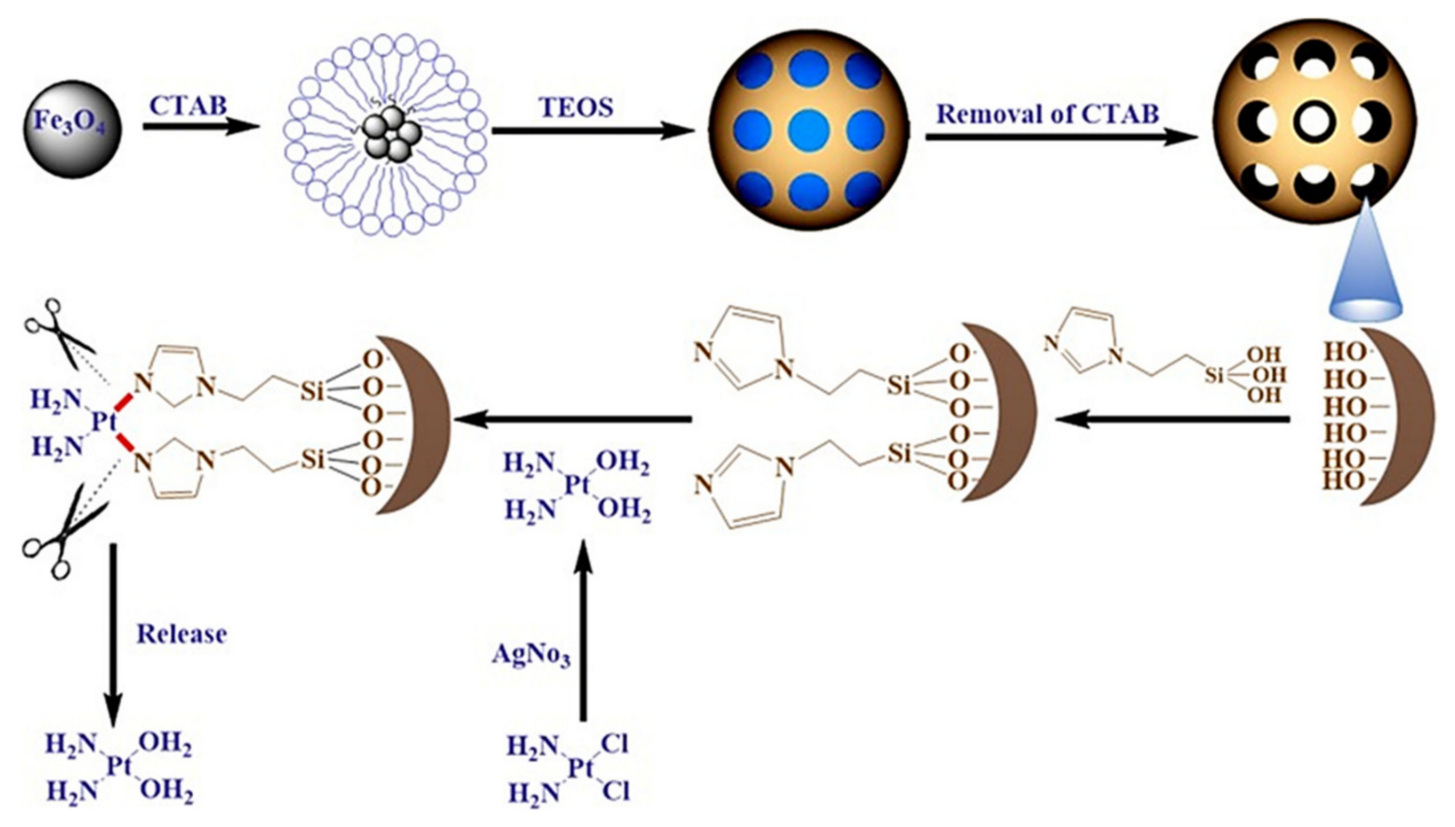
Synthesis of magnetic mesoporous silica nanoparticles functionalised with triethoxy-3-(2-imidazoline-1-yl)propylsilane and release of platinum drug [49].

Mesoporous magnetic silica nanoparticles were described as novel system for magnetic resonance imaging and application in cancer chemotherapy. Their surface was decorated with imidazoline functional groups that allowed conjugation with cis-diaquadiamino platinum(II) [49] (Figure 10).

Fe_3_O_4_ nanoparticles were made from FeCl_2_·4H_2_O and FeCl_3_·6H_2_O and stabilized with calcium alcoholic solution (CA). Fe_3_O_4_-CA nanoparticles were then covered with silica shell using TEOS in the presence of pore generating n-hexadecyltrimethylammonium bromide (CTAB). Mesoporous particles were then functionalized with imidazoline groups and conjugated with Pt(II) derivative, generated in situ from cisplatin. The A2780-cell viability MTT assay revealed that the nanoparticles were as effective as cisplatin itself, though slightly more effective at higher concentrations (100 µg/mL). The calculated IC_50_ values were calculated as 1.19 and 1.23, respectively, for cis-Pt and the cis-Pt containing mesoporous system.

**Figure 11:**
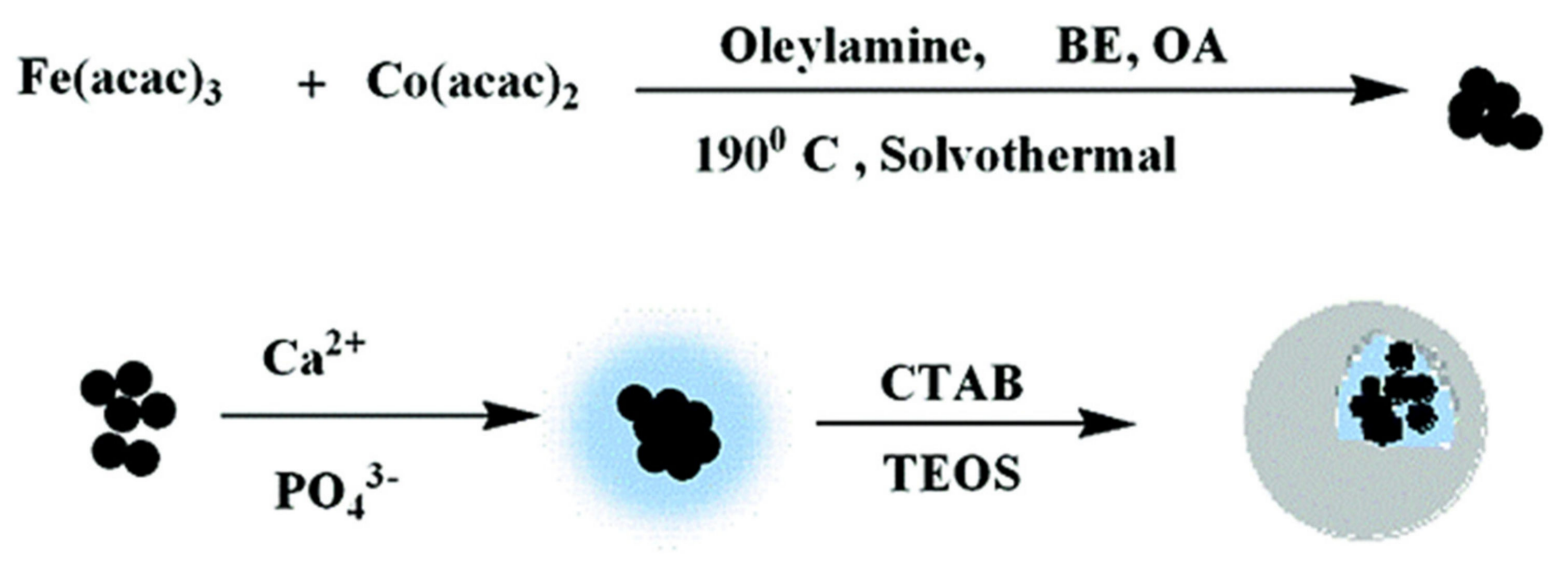
Synthetic scheme of CoFe_2_O_4_ encapsulating silica NPs [50].

**Figure 12:**
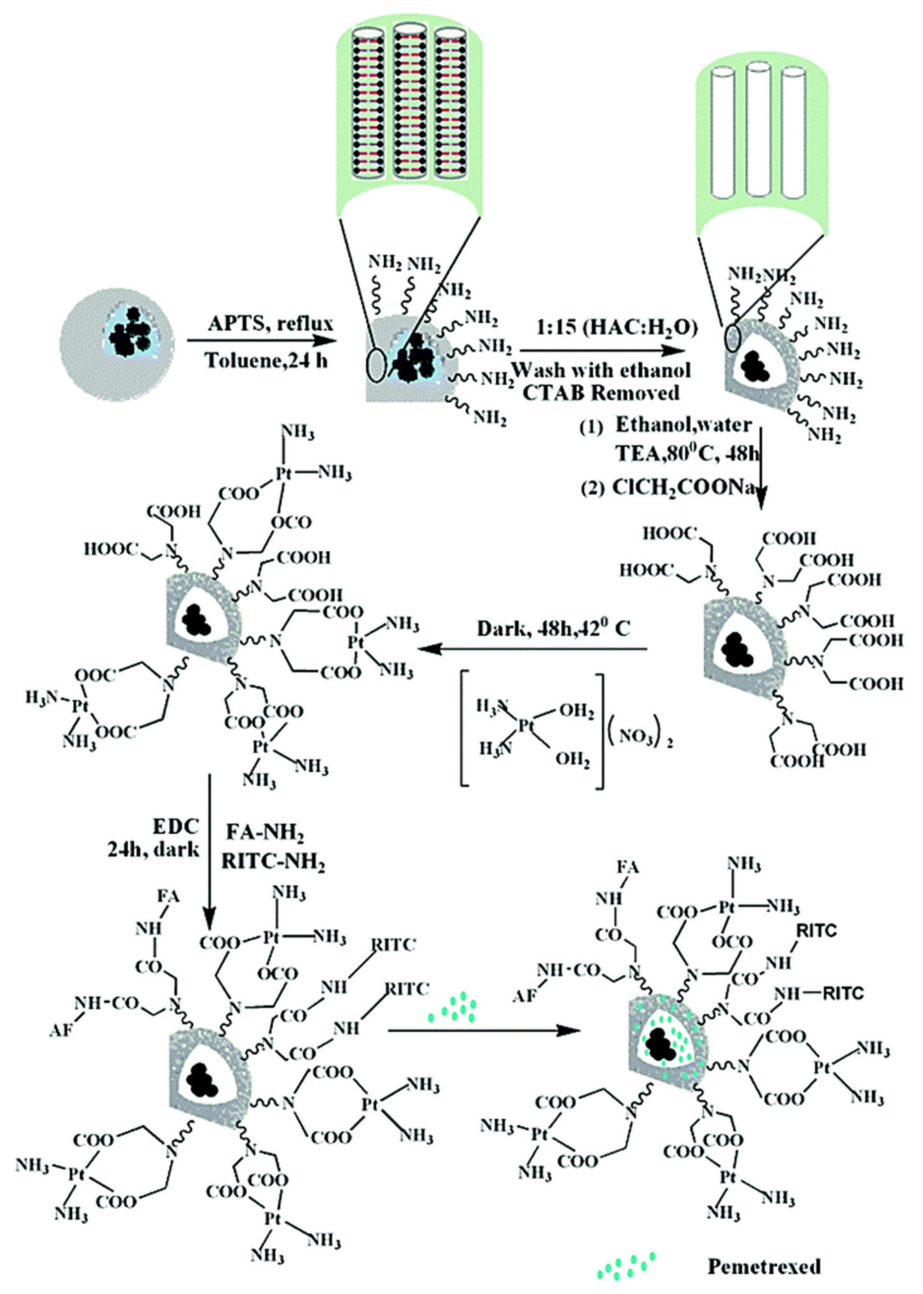
Functionalization of silica nanoparticles with iminodiacetic acid and the conjugation with Pt-drug, FA and RITC [50].

A multifunctional mesoporous hollow silica nanocapsules with conjugated cis-diaquadiamino platinum(II) and additionally loaded with permetrexed was synthesized and equipped with folic acid (FA), and rhodamine isothiocyanate (RITC) moieties. The synthetic approach is presented in detail in Figure 11 and Figure 12. The internal space was accommodating hydrophobic drug—permetrexed and superparamagnetic CoFe_2_O_4_ while the external one was grafted with Pt drug, FA and RITC. The cytotoxicity studies have shown that the dual drug nanocarrier system exhibits enhanced effect compared to individual drugs as a result of drug synergism and site targeting through folate receptor [50].

**Figure 13:**
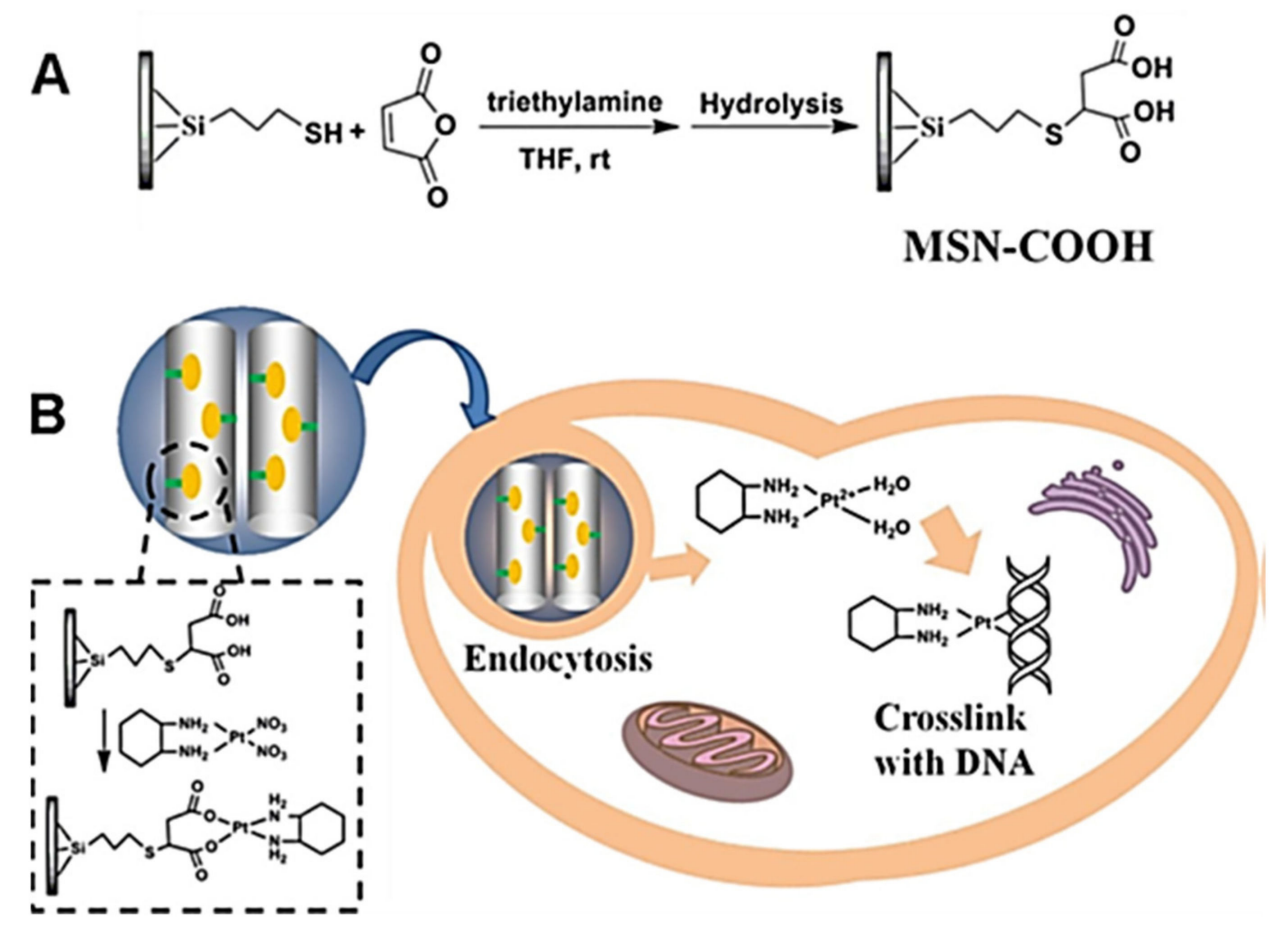
Modification of 1,2-bidentate carboxyl groups onto the parent nanoparticles of MSN-SH via thiol-ene click reaction (**A**); MSN nanoparticles conjugated with the oxaliplatin active species and the intracellular release of platinum drugs via acid mediated hydrolysis to inhibit the replication of DNA (**B**) [51].

Mesoporous nanoparticles were also exploited as carrier system for conjugation of different platinum drug—oxaliplatin, serving improved anticancer drug delivery [51]. The nano-carriers with 1,2-bidentate carboxyl groups were synthesized and reacted with 1,2-diaminecyclohexano platinum(II) dinitrate, as shown in Figure 13. MSN-Pt system was found to exhibit an improved cytotoxicity against HepG-2 cells, when compared to that of free oxaliplatin (Table 1).

**Figure 14:**
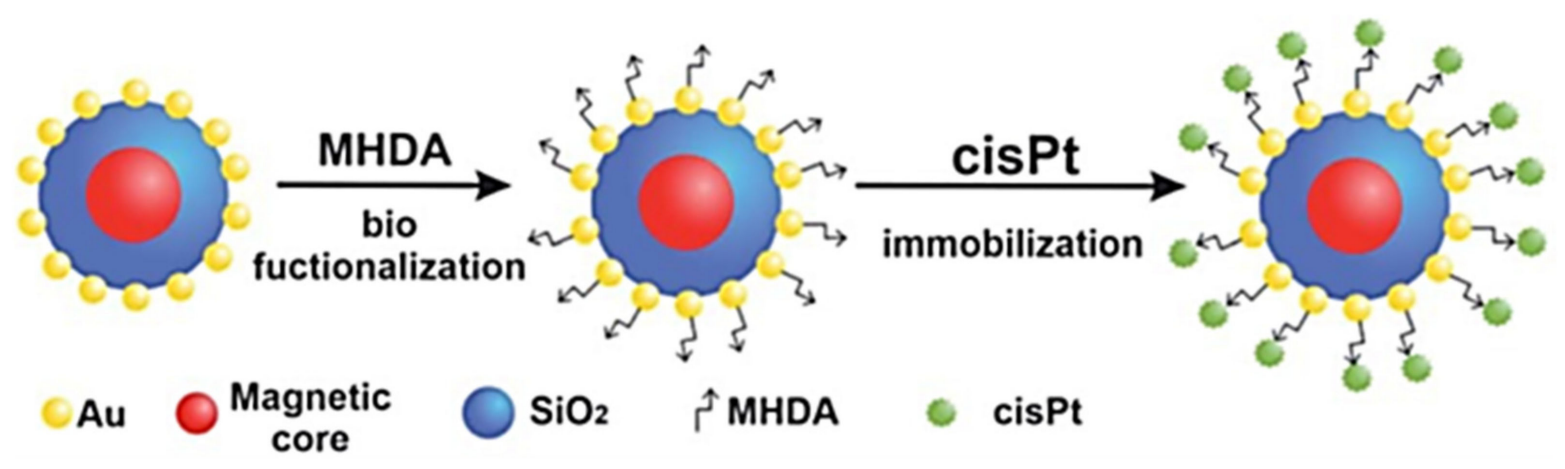
Scheme of the Fe_3_O_4_@SiO_2_@Au NPs biofunctionalization process and process of the cPt immobilization [52].

Direct cisplatin immobilization on Fe_3_O_4_@SiO_2_@Au nanoparticles was described recently. Hydrophobic Fe_3_O_4_ NPs were prepared in a two-step thermal decomposition process via intermediate iron-oleate complex. Then the Fe_3_O_4_@SiO_2_ system was made by reverse microemulsion method by constructing outer shell using TEOS and APTES and final decoration of the nanoparticles with 2–6 nm Au particles. Subsequently 16-mercapto-hexadecanoic acid (MHDA) linker was applied to link with the NPs surface and immobilize cisplatin via COOH group (Figure 14) [52].

**Figure 15:**
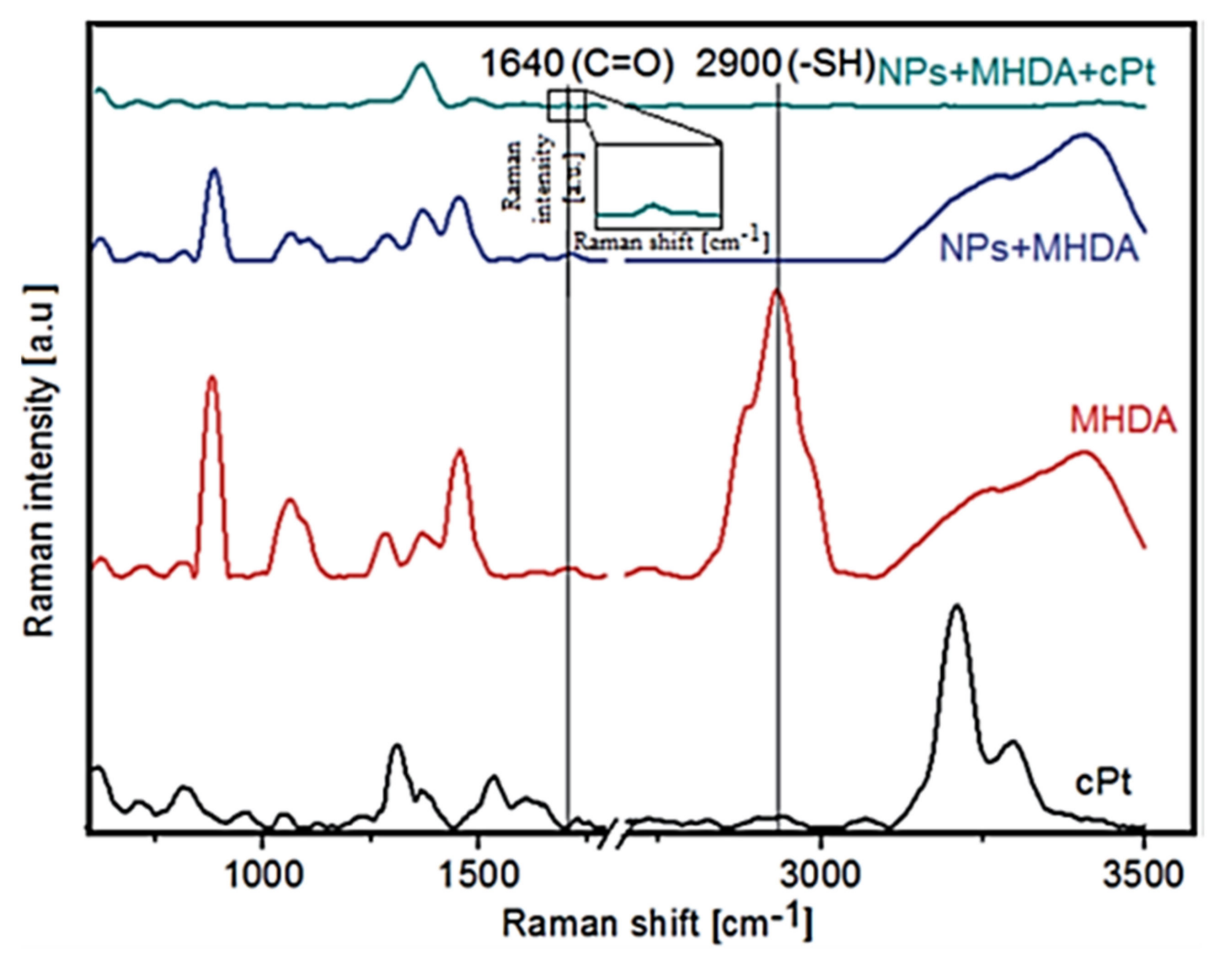
Raman spectra of: cisplatin (black spectrum), the pure MHDA (red spectrum), the Au NPs + MHDA (blue spectrum), Fe_3_O_4_@-SiO_2_@Au + MHDA + cisplatin (green spectrum). There is a mistake in the figure as the value for C=O differs from the one in the text in both papers [52,53].

There are, however, weak proofs for actual chemical linking of cisplatin with –COOH moiety of MHDA. The authors claim that the absorption band of C=O at 1680 cm^−1^ shows linking of cisplatin with NPs surface, but the absorption of free carboxyl group lies in exactly the same region of 1725 ± 65 cm^−1^. So there seems to be a real possibility that cisplatin is simply entangled physically within MHDA (Figure 15) [52,53].

The authors claim that the compact (below 40 nm) nanoparticles can serve as potential system in magnetic resonance imaging guided chemo-photothermal treatment of colon cancer cells SW480 and SW620. The estimated viability reduction was in the range of 40–50%. One has to mention that, much earlier [54], there was a report on covalent bonding of cisplatin to carboxyl groups. However, no significant proof was presented, though ^195^Pt NMR could be conclusive in such cases.

### 2.3. Silsesquioxanes-Platinum Drugs Conjugates

**Figure 16:**
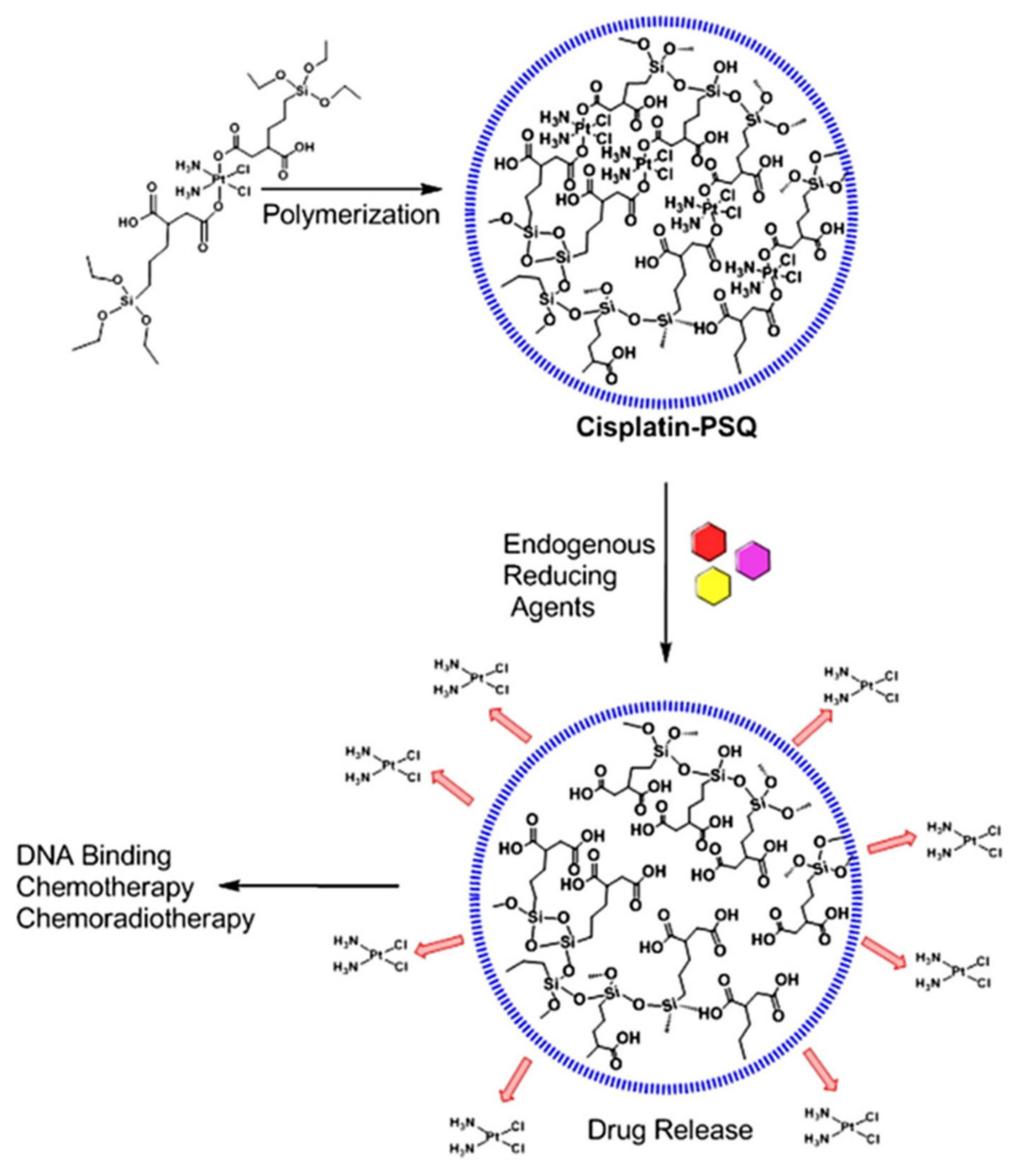
Synthetic and release scheme for cisplatin-polysilsesquioxane system [18].

There has been a considerable attention drawn to the applications of POSS containing drug conjugates, since POSS is regarded as the next generation of materials in biomedical fields [19,55,56,57]. Compared to functionalized silica as drug delivery system, silsesquioxanes present much more uniform size (~1.5 nm). They have been found to be non-toxic themselves, while even simple co-delivery with POSS allows for more effective penetration of drugs through cell membranes [55,58,59]. In light of the above, it appears that the literature concerning POSS-platinum drugs conjugates is relatively scarce [60,61]. However, it is worth presenting the approach to polysilsesquioxane nanoparticles crosslinked by platinum(IV) prodrug (Figure 16) [18,62]. The authors presented an interesting synthetic pathway, though their conclusion that Pt containing nanoparticles demonstrate higher therapeutic efficacy than cisplatin itself is not fully supported by results. The in vitro tests against A549 and H460 lung cancer cells revealed that the IC_50_ for the conjugate was 14.9 µm against A549 and 2.07 µm against H460 cells compared to 3.5 µm and 0.65 µm respectively for cisplatin. Slightly higher efficacy of nanoparticles over cisplatin was found only in in vivo studies once chemoradiotherapy approach was applied.

## 3. MSN Complexes with Platinum Drugs

### 3.1. Cisplatin (Pt(II)) Organosilicon Nanocomplexes

**Figure 17:**
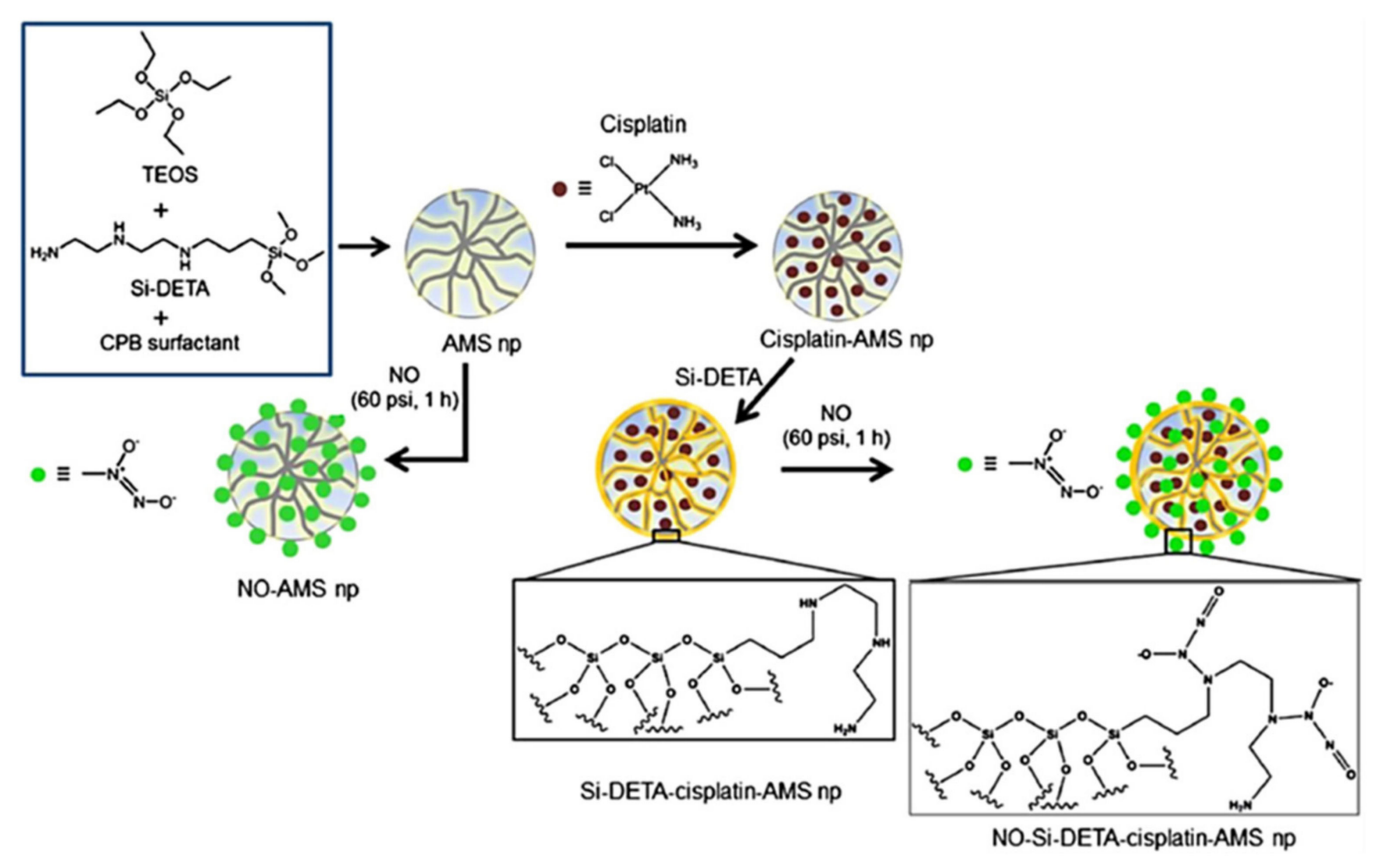
Preparation of nitric oxide-cisplatin loaded amine-functionalized mesoporous silica [64].

Paqua, Balkus et al. used mesoporous silica nanoparticles coated with DOPC lipid as a platinum drugs carrier (cis-platin, carboplatin and oxaliplatin) for combined chemo and radiotherapy [63]. One year later, they published results on mesoporous silica nanocarrier, releasing nitric oxide (NO) and cisplatin for the treatment of non-small cell lung cancer (NSCLC) [64] (Figure 17). Promising results were obtained in the case of NSCLC cell lines (i.e., H596 and A549) for which the toxicity of silica nanoparticles loaded with cisplatin and NO (NO-Si-DETA-cisplatin-AMS) was significantly higher, compared to silica nanoparticles loaded with cisplatin only (Si-DETA-cisplatin-AMS). Amine-functionalized mesoporous silica (AMS) nanoparticles were synthesized by condensation of tetraethylorthosilicate (TEOS) and 3-triethoxysilylpropyl)diethylenetriamine (Si-DETA) in the presence of CPB (cetylpyridine bromide) surfactant. Spherical nanoparticles with an average size of approx. 50 nm were obtained, containing micro and mesopores of 2–8 nm. NO was used to increase the therapeutic index, allowing for the use of lower doses of the drug in therapy. There was a number of reports on the positive effect of using NO in the treatment of cancer [65,66], also in the case of silicon nanoparticles nano-carriers [67].

**Figure 18:**
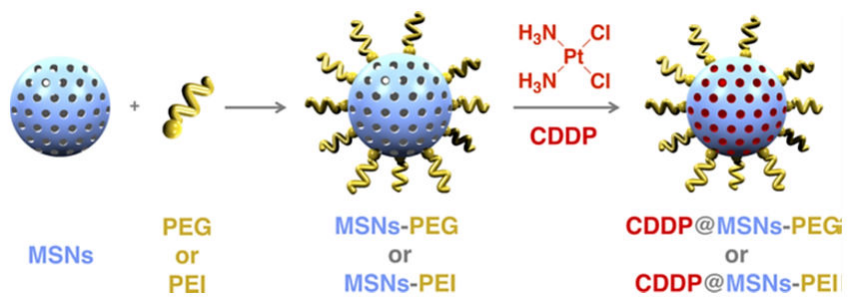
Platinum loaded mesoporous SiO_2_ modified with PEG and PEI [68].

**Figure 19:**
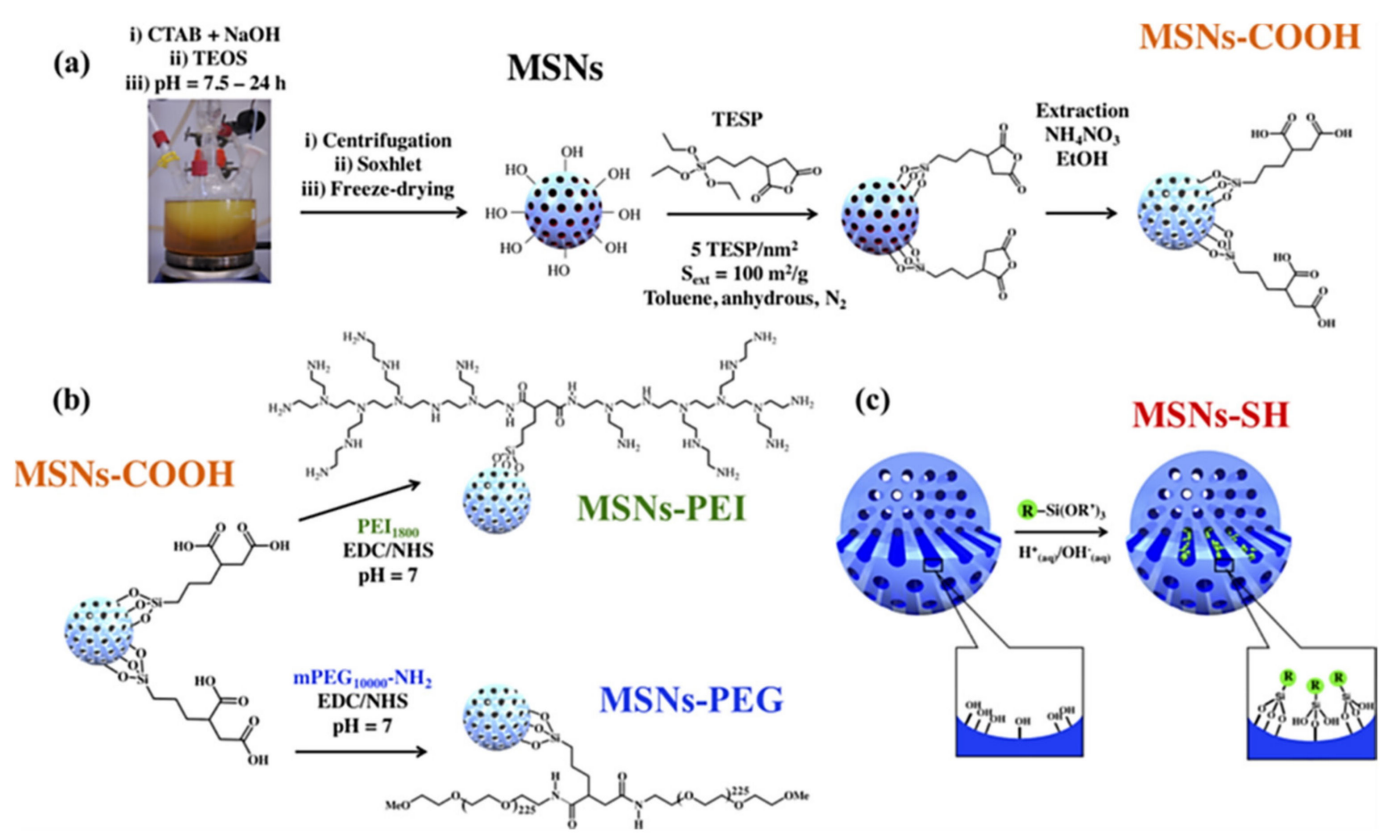
Modification of MSNs via (**a**) surface grafting of carboxylic groups (MSN-COOH) by postsynthetic modification with TESP, (**b**) coating of PEG and PEI by amidation (**c**) co-condensation with MPTES leading to MSN-SH. R = −(C_3_H_6_)SH and R′ = −(OCH_2_CH_3_)_3_ [69].

Nanoporous silica was also functionalized with poly(ethylene)glycol—PEG (Mw = 10,000 g/mol) or low molecular weight (Mw = 1800 g/mol) branched polyethyleneimine—PEI and successfully used as cisplatin carrier [68] (Figure 18). Bouyer et al. obtained nanoparticles with the size of approximately 140 nm by covalent grafting method involving the reaction of the MSN hydroxyl groups with TESP, later the formed carboxyl groups were reacted with the amine groups of the polymers (PEG or PEI). The modification increased biocompatibility of the drug-carrier system, reduced the undesirable effects of nanoparticles build-up in lungs, kidneys and increased the half-life of the drug [69]. The high porosity and colloidal stability in aqueous solutions of the resulting functionalized nanoparticles made them suitable for drug delivery applications. The strong affinity of sulfur for platinum was also taken into account. Therefore, MSN functionalized with thiol groups embedded in the pores of nanoparticles were synthesized. Mercapto-functionalized mesoporous silica nanoparticles (MSN-SH) were prepared by co-condensation of (3-mercaptopropyl)triethoxysilane (MPTES) and triethoxysilane TEOS. As a result of introducing thiol groups into mesoporous silica, nanorods were formed with a diameter of 70 nm and a length of 327 nm (Figure 19). Taking into account the length of cisplatin release from the three tested carriers (MSN-PEI, MSN-PEG and MSN-SH), it was shown that PEI-functionalized MSN nanoparticles were the most promising material in anticancer therapy. Attention to the method of drug loading was also drawn and in order to avoid undesirable reactions of cisplatin with DMSO [70], the solvent was eliminated in synthetic approach.

**Figure 20:**
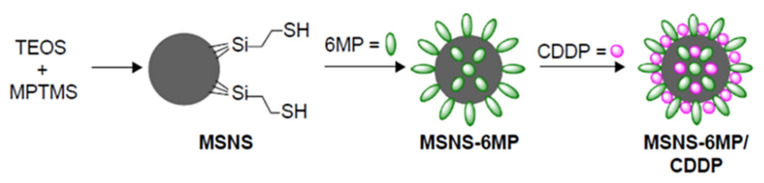
Schematic synthesis of MSNS-6MP-cisplatin [71].

Zhao et al. applied a combination strategy by covalent attachment of 6-mercaptopurine (6MP) to the surface of MSN and loading cisplatin into the pores of modified silica carrier [71], containing thiol groups (Figure 20). The contents of cisplatin in the MSN-6MP-cisplatin system was 100 mg/1 g (1 g of MSNS-6MP-cispltin contained 100 mg of cisplatin). The in vivo studies using the S180 mouse model revealed that MSNS-6MP-cisplatin system was significantly more effective in chemotherapy than the classical combination of 6MP plus cisplatin it also completely eliminated the serious renal and cardiac toxicities induced by cisplatin.

**Figure 21:**
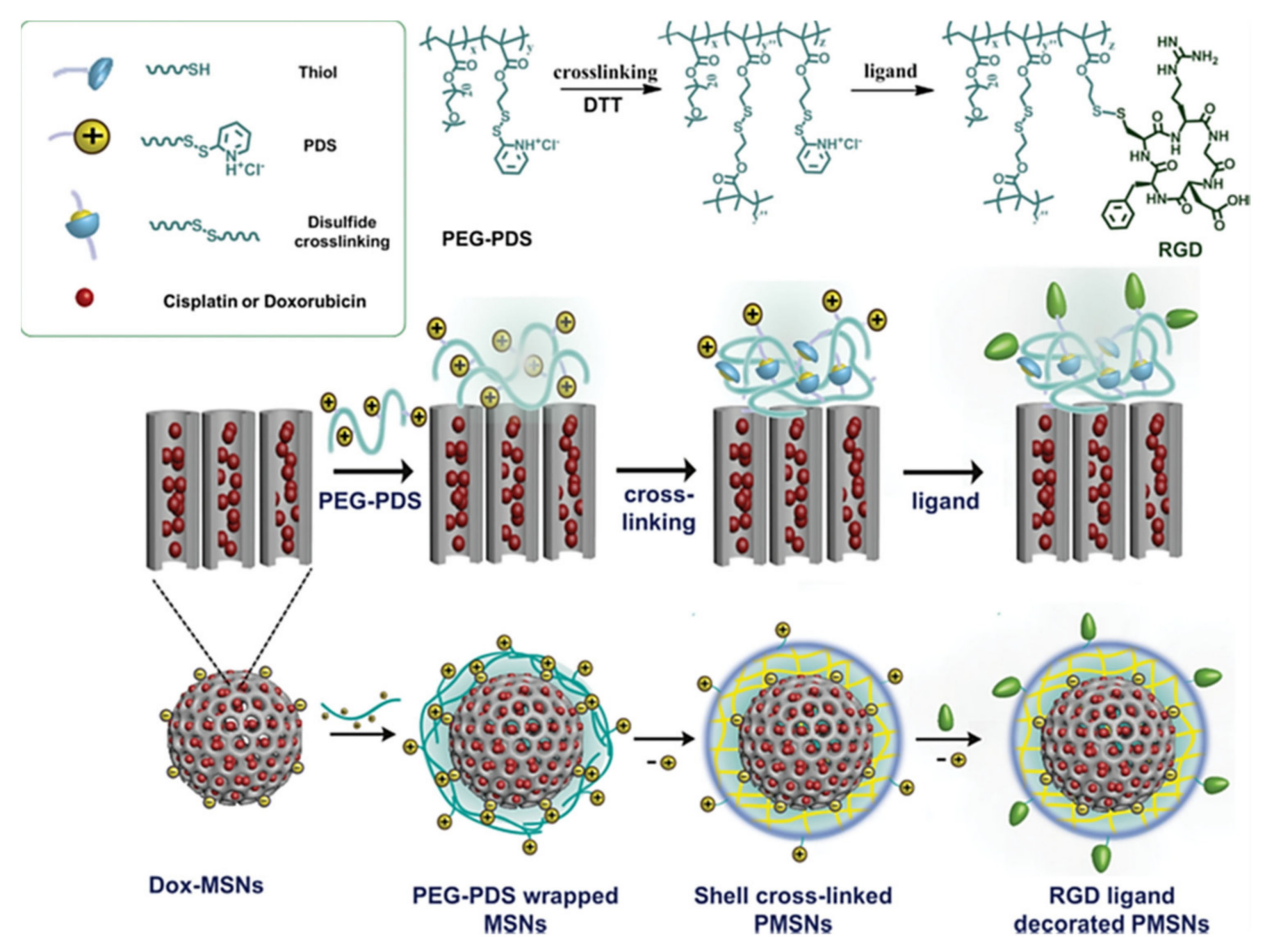
One-pot synthetic pathway of polymer-gatekeeper MSN with stimuli responsive polymer (PEG-PDS) and targeting ligand (cRGDfC) [72].

A one-pot synthesis of an MSN system that can encapsulate cisplatin with high drug loading of 33 wt% (alternatively 44 wt % of doxorubicin) was described. The pores of MSN containing drug were non-covalently end-capped with a polymer gatekeeper—copolymer containing pyridine disulfide hydrochloride (PDS) and poly-ethylene glycol (PEG) as side chains. PEG on the surface of MSNs provides water solubility, while PDS facilitates wrapping of the MSN surface through the weak electrostatic interaction between the MSN and the polymer [72] (Figure 21). A targeting agent—cyclic peptide (Arg-Gly-Asp-D-Phe-Cys) (cRGDfC) was attached to the surface of this system by disulfide bond. Drug molecules could be effectively loaded into MSN and assured stable location inside the pore due to blocking of pores with a stimulus-responsive polymer. However, the cytotoxicity analysis for cisplatin-loaded system at 48 h in KB cells and concentration of the drug of 2 µg/mL showed cell viability of ~35%. It was lower by 10% once the MSN system contained two drugs, cisplatin and doxorubicin respectively at the concentrations of 1.4 and 2 µg/mL.

**Figure 22:**
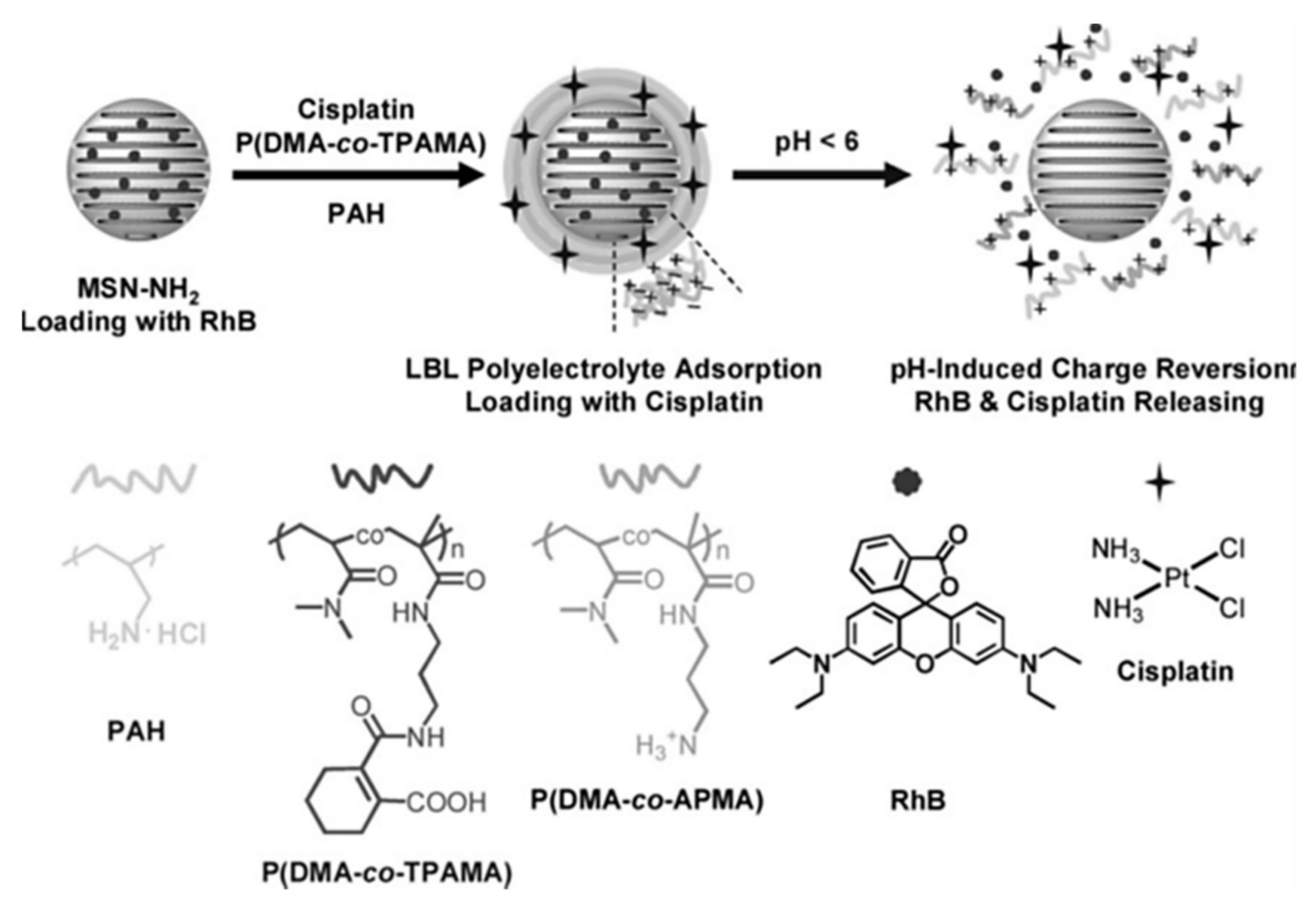
Preparation of organic/inorganic hybrid MSN coated with P(DMA-co-TPAMA)/PAH polyelectrolyte multilayers and the pH-triggered dual release of cisplatin adsorbed within multilayers [73].

The use of polymers to create cisplatin delivery system based on silica nanocarrier that was capable of triggered co-release of cisplatin and model drug molecules [73] was demonstrated. The important role of polyelectrolytes in the distribution and release of drugs from the pores of MSN at reduced pH was underlined. The developed system (Figure 22) included the outer polyelectrolyte multilayer constructed of cationic poly(allylamine hydrochloride) (PAH) and negatively charged P(DMA-co-TPAMA) consisting of 3,4,5,6-tetrahydrophthalic anhydride functionalized N-(3-aminopropyl) methacrylamide (TPAMA)) and N,N-dimethylacrylamide (DMA). Cis-platin was incorporated into the layer of polyeteltrolytes to form complexes with P(DMA-co-TPAMA). Additionally, a model drug (rhodamine, RhB) was placed in the pores of MSN. It was suggested that rhodamine could be replaced with anticancer drugs such as doxorubicin or paclitaxel. Since the release of cisplatin and RhB from such the system can be achieved at pH 5–6, while being quite slow at pH 7.4, the developed system is expected to be effective in vitro.

**Figure 23:**
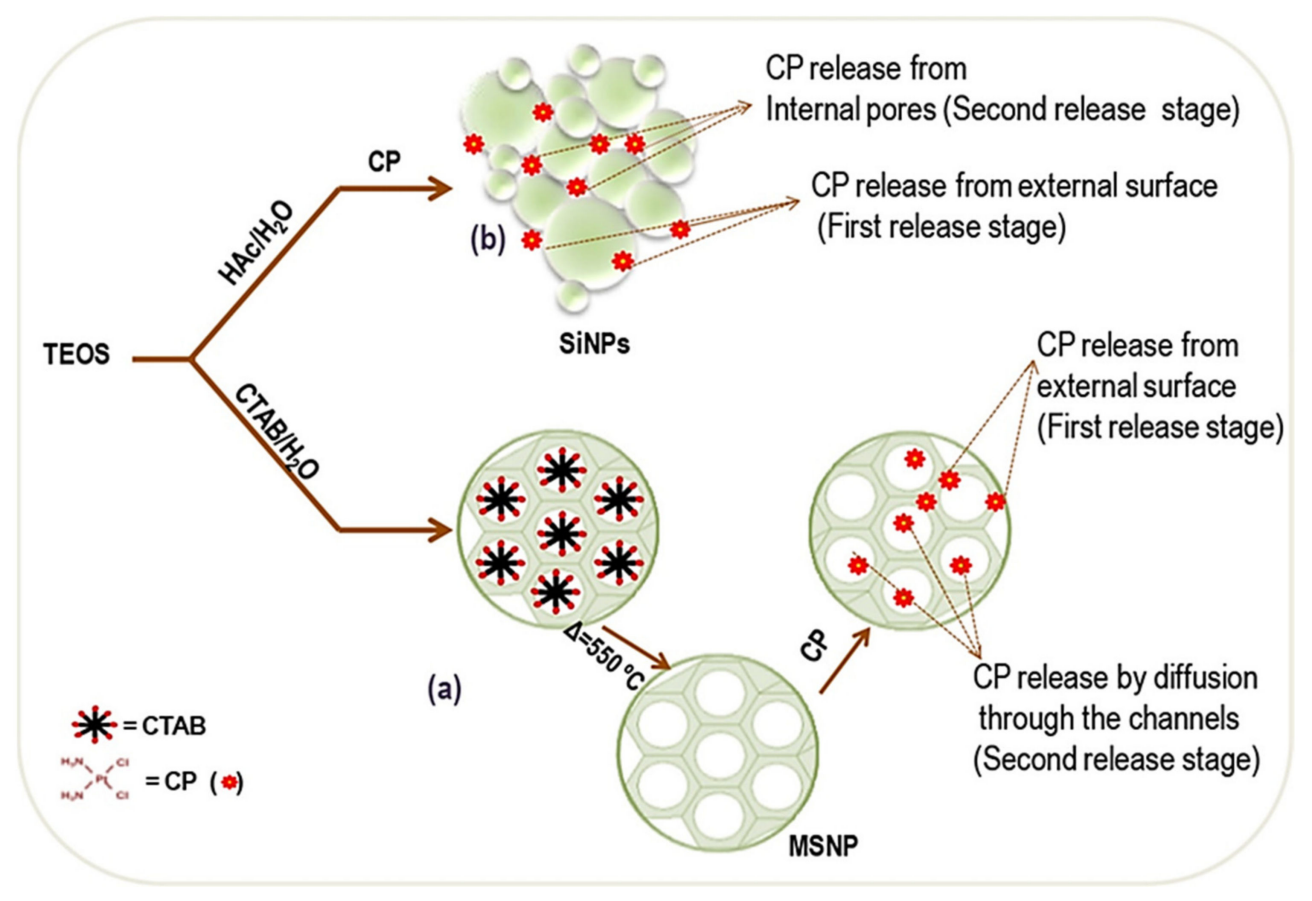
Scheme of cisplatin loading and CP release [74].

Recently, the effectiveness of two different types of silica nanostructures as carriers for the release of cisplatin was analyzed [74]. The so-called MSNP-cisplatin system was prepared using classic methodology based on CTAB as a template, reported earlier. The other type of silica nanoparticles (SiNP)—agglomerates was obtained in a presence of acetic acid as a catalyst of TEOS hydrolysis-condensation (Figure 23). Both silica based materials were loaded with cisplatin and were found to be biocompatible. They have shown a fast release of cisplatin, followed by a slow one. Cisplatin was released faster from MSNP than from SiNP nanocarrier tetraethoxysilane (TEOS) hydrolysis-condensation reaction [75]. However, the cell viability against C-6 cells was, after 24 h, in the range of 60% for SiNP-cisplatin (5000 µg/mL) and 70% for MSNP-cisplatin (1000 µg/mL).

### 3.2. Complexes with Other Platinum Drugs

**Figure 24:**
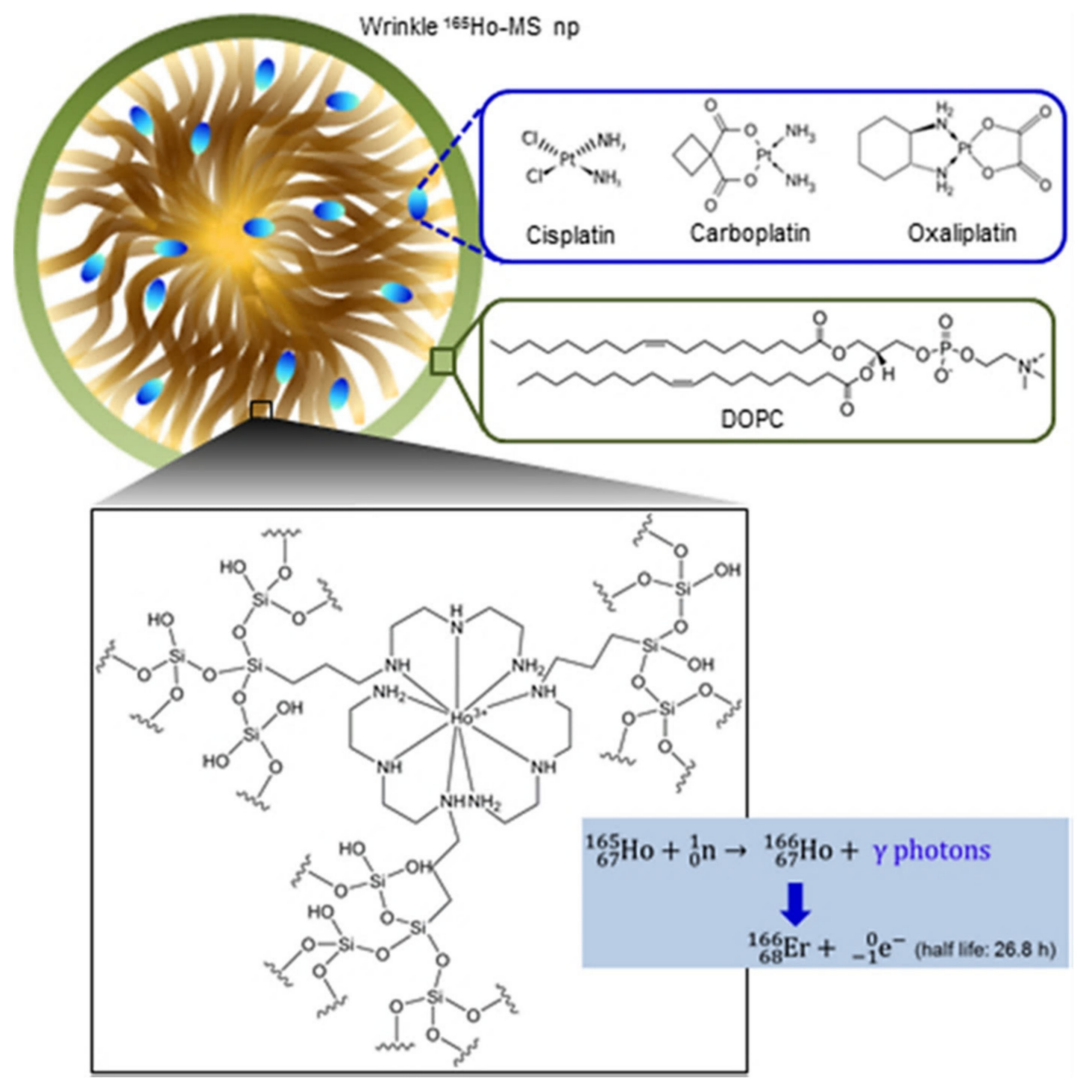
Platinum drugs loaded holmium-165-containing, wrinkled mesoporous silica nanoparticles DOPC lipid coated [63].

The currently approved platinum drugs (called often platins) include, apart from cisplatin, also carboplatin, oxaliplatin and nedaplatin. A novel chemoradiotherapeutic 1,2-dioleoyl-sn-glycero-3-phosphocholine lipid coated/uncoated platinum drug loaded (carboplatin, oxaliplatin, cisplatin) and radioactive ^166^Ho-containing, wrinkled mesoporous silica nanoparticles have been described [63] (Figure 24). The synthesis of such the system involved formation of a microemulsion using cetylpyridinium bromide (CPB) as a template and urea. Due to the latter silica precursor (TEOS) and ^165^Ho-SiDETA were hydrolyzed, and then self-assembled. Wrinkled nanoparticles were formed having large surface areas due to the fibrous morphology. In the next step, platinum drugs were adsorbed on the carrier surface.

Platinum drugs loading was respectively 14.6, 11.7, and 16.1% (*w*/*w*) for cisplatin, carboplatin, and oxaliplatin. The additionally stabilized particles were also prepared by the use of DOPC lipid, forming a coating with a thickness of 7–10 nm and average size of the entire system ~95 nm. The choice of lipids in this system was justified by increased biocompatibility and the ability to mimic cell membranes, facilitating drug transport through membrane channels, but lipid-coated systems showed a slower release of platinum drugs when compared to the uncoated ones.

**Figure 25:**
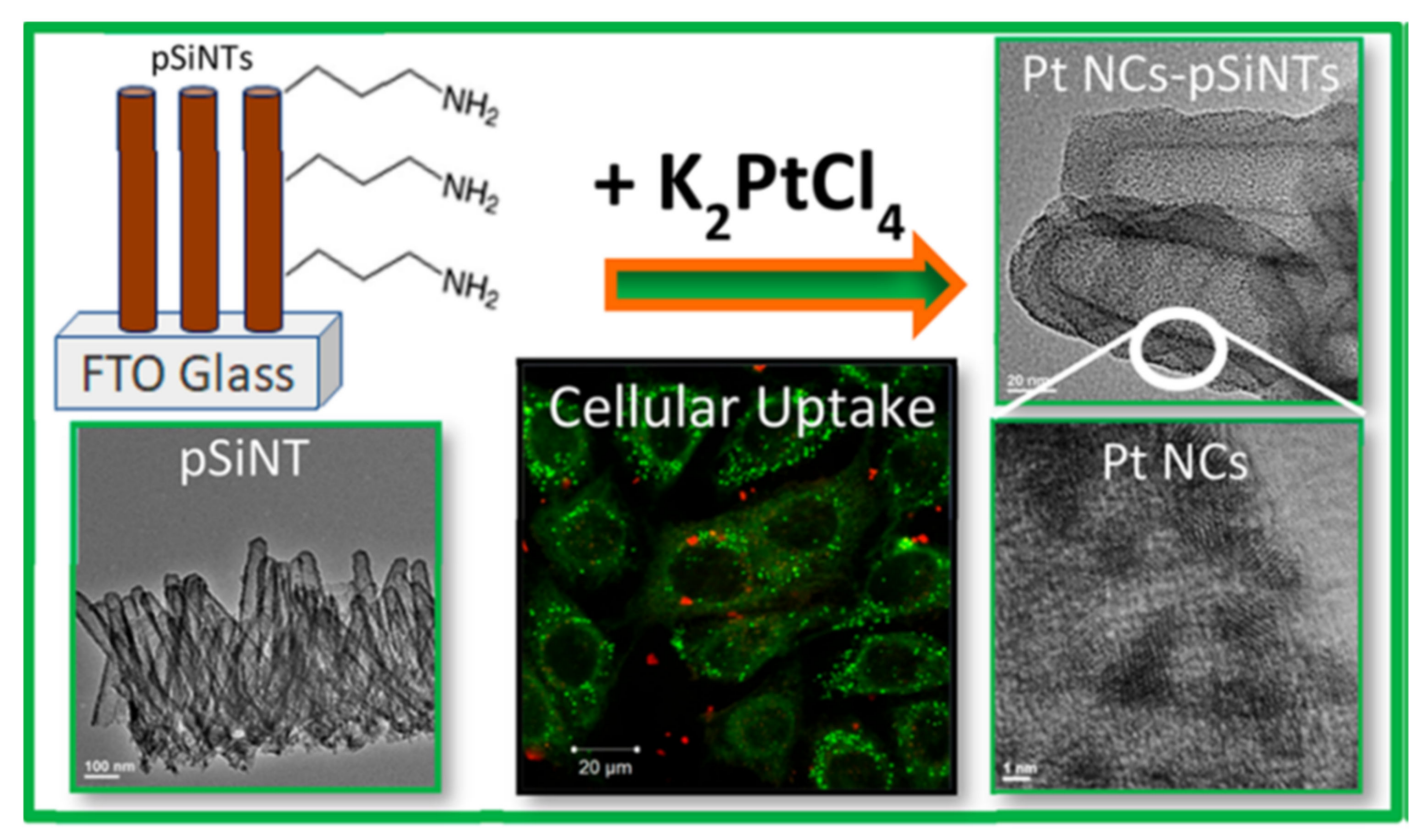
Pt NCs-pSiNTs structure [76].

**Figure 26:**
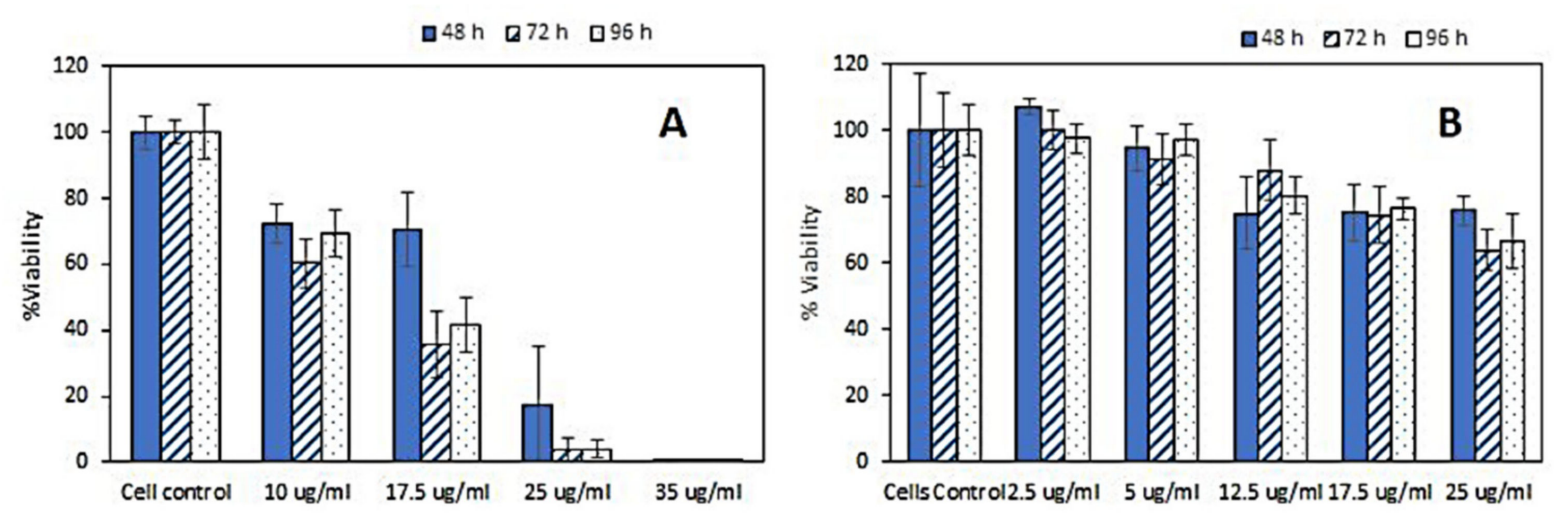
Cytotoxicity of (**A**) Pt NCs (3.5 ± 1.1 nm) and (**B**) K_2_PtCl_4_ at different doses [76].

One of the novel interesting strategies is targeted delivery of platinum nanocrystals (Pt NCs)—caged Pt nanoclusters using APTES modified biodegradable porous silicon nanotubes (pSiNTs) as templates. The Pt NCs support (35–50 nm) was suitably functionalized with 3-aminopropyltriethoxysilane (APTS) to obtain amine functions and its surface was then coated with the Pt NCs nanocrystals (1–3 nm) by incubating the carrier with K_2_PtCl_4_ [76] (Figure 25). The in vitro studies showed that such the constructed system exhibited strong toxicity towards cervical cancer (HeLa) cells, by inducing apoptosis as compared to K_2_PtCl_4_ (Figure 26).

**Figure 27:**
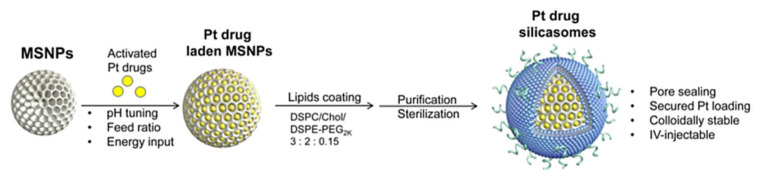
Synthetic steps for preparation of silicasomes incorporating platinum drugs [77].

Currently, a facile and effective platinum drugs loading method was presented allowing for delivery by a silicasome nanocarrier, comprising of a mesoporous silica core surrounded by a lipid bilayer. It encapsulated drugs inside the pores as well as inside the bilayer itself [77] (Figure 27). The preparation of such systems was performed for three properly activated cationic platinum drugs generated from oxaliplatin, cisplatin and dichlorobis(ethylenediamine)platinum (Pt(en)Cl_2_ in reaction with AgNO_3_. The in vitro and in vivo studies against KPC pancreatic adenocarcinoma model were performed with silicasome developed from oxalitplatin. It have shown improved PK profile and intratumoral drug delivery over free drug together with significant reduction in bone marrow toxicity.

### 3.3. Targeted Therapy

Targeted delivery of drugs called also active drug delivery exploits the presence of specific receptors on the surface of target cells, due to which it is possible to precisely deliver drugs to tumor cells and avoid systemic toxicity [78,79]. In this therapy, a number of targeting agents is applied with affinity for tumor cell receptors—the most widely used and inexpensive are folic acid, vitamin B_12_ or biotin [80,81].

**Figure 28:**
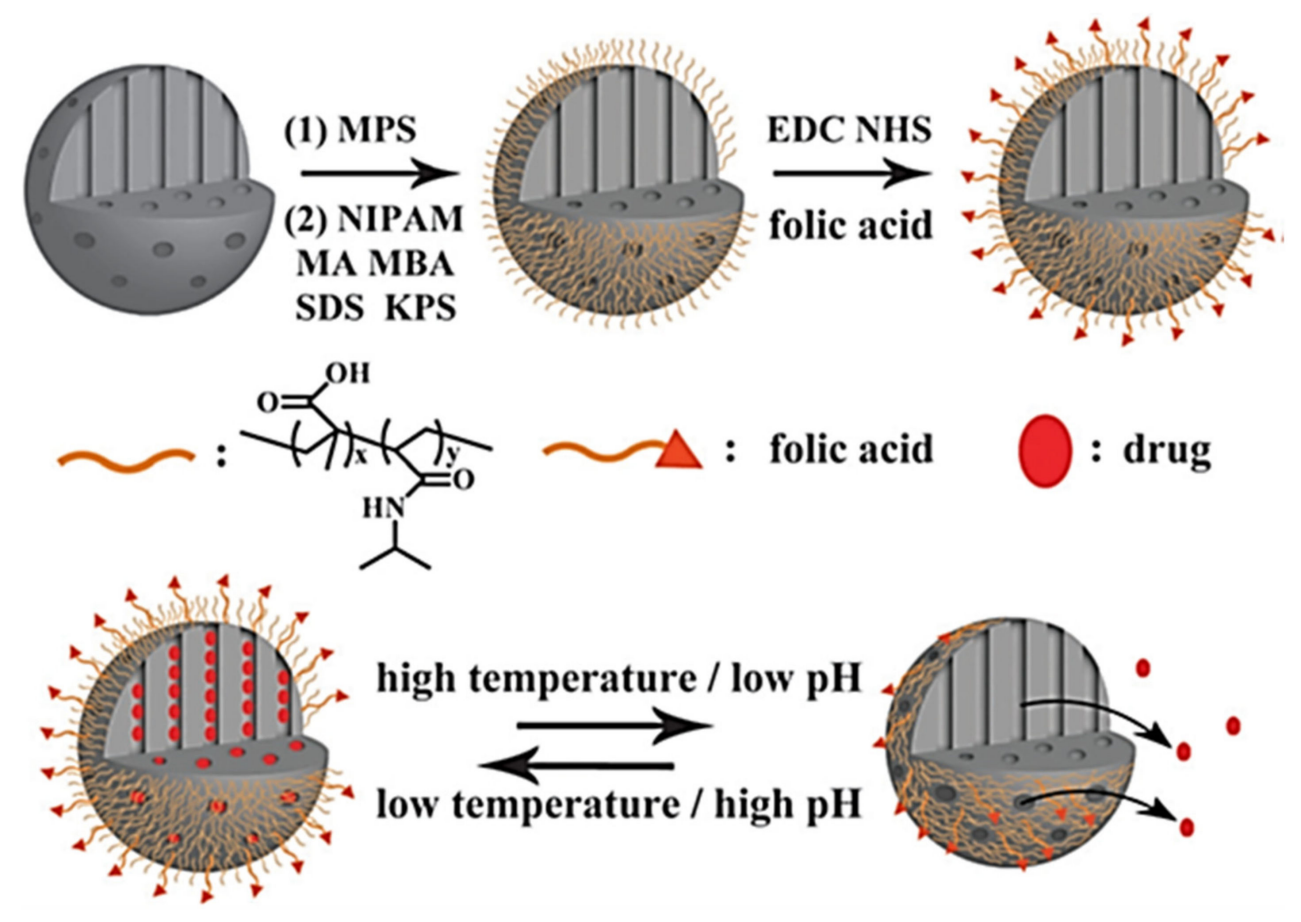
Preparation of silica nano-carrier with targeting agent and release of cisplatin [83].

A system based on the use of folic acid as a targeting agent (Figure 28) was proposed by Jin, Song Liu et al. [82]. MSN were prepared by the base-catalyzed sol-gel method with the use of CTAB, (diameter of 100 nm pore diameter of ~2.5 nm) and a specific surface area of 786 m^2^/g. The surface of the nanoparticles was coated with temperature and pH sensitive poly[(N-isopropylacrylamide)-co-(methacrylic acid)] [83] to slow down the release loaded cisplatin from the MSN pores. Folic acid molecules were covalently grafted to nanocarriers by the reaction between the COOH groups of MSN@p(NIPAM-co-MA) and NH_2_ groups on the FA molecules to target Hep2 laryngeal squamous cell carcinoma cells containing folate receptors. In contrast, cisplatin was loaded inside the pores of MSN. This cisplatin delivery targeting system was ~ twice more effective towards HeLa cell line (viability of 20% after 24 h, at 60 µg/mL) compared to free cisplatin and the system without targeting agent. Additionally it showed significantly enhanced release at higher temperatures and lower pH.

Another nano-carrying system, including folic acid targeting agent was recently described [84]. Its effectiveness in killing glioblastoma cancer cells (LN-18) was analyzed. Mesoporous silica nanoparticles were synthesized by Stöber method with the use of CTAB to obtain uniform spherical nanoparticles with the size below 100 nm, larger surface area (1011 m^2^/g) and larger mesopores (4.35 nm). The authors claim that these parameters affected the long-term release profile of cisplatin. Folic acid was chemically anchored to the silica nanoparticles surface by a carbodiimide reaction. However, the IC_50_ values were lower for Cis-Pt than for MNPSiO_2_-FA/Cis-Pt, 78.6 µg/mL, and 149 µg/mL, respectively.

**Figure 29:**
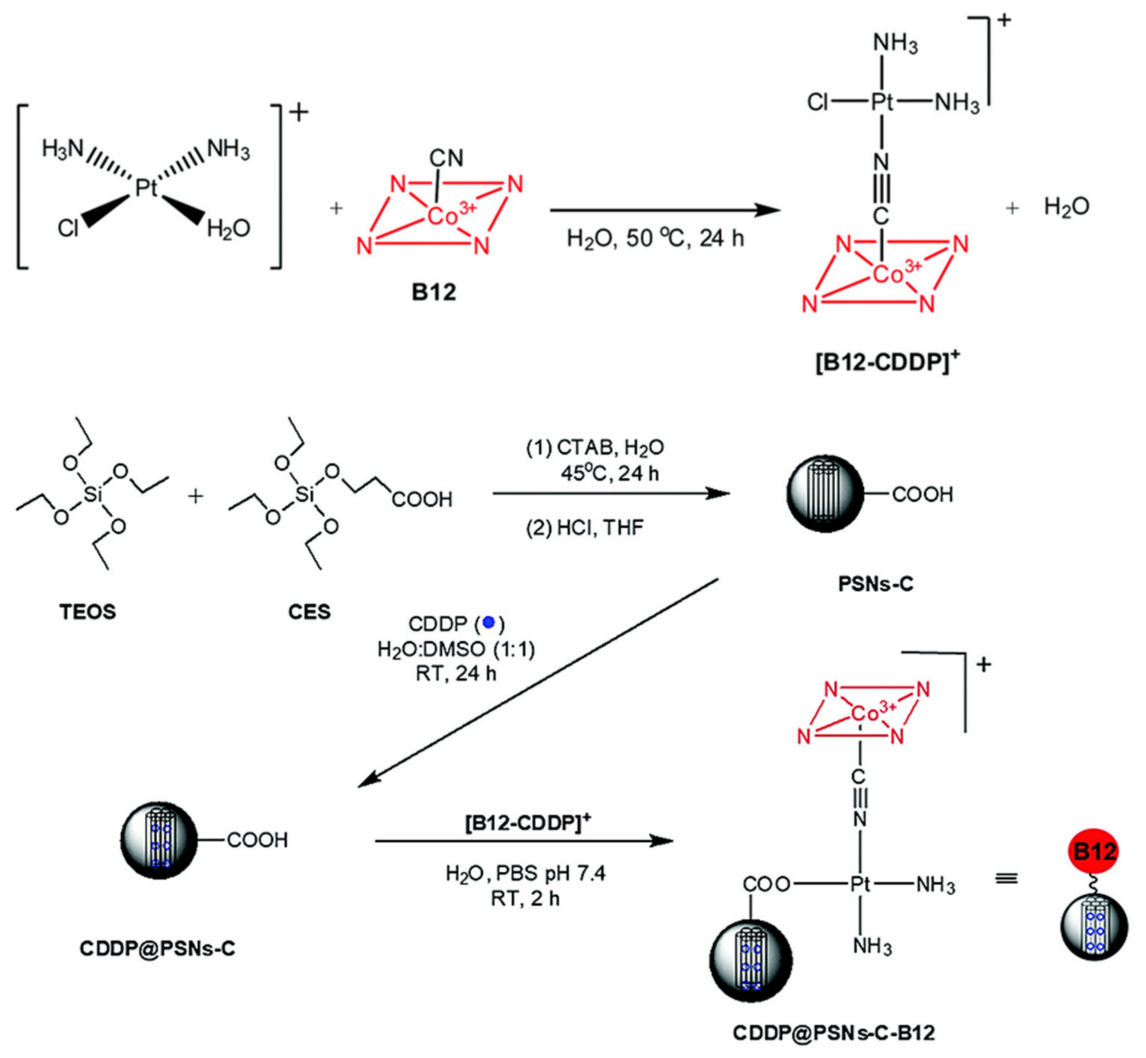
Synthetic scheme for preparation of CDDP@PSNs-C-B_12_ nanoparticles [85].

A novel targeted cisplatin delivery system based on B_12_-conjugated silica nanoparticles was also described (Figure 29) [85]. The system design involved carboxyl group-modified PSNs (PSNs-C) as a drug carrier, B_12_ as a targeting molecule, and cisplatin (CDDP) as an anticancer drug. PSNs-C were synthesized by co-condensation of TEOS and (carboxyethyl)triethoxysilane (CES) using CTAB as the porous template. This nanocarrier had carboxylic bonds and loaded cisplatin. The latter had the ability to coordinate with the cobalt center of vitamin B_12_. The vitamin also served in hindering early release of the drug in a reducing environment (at pH of 5.5), the cobalt center is reduced and detached from the carrier, which causes the release of cisplatin at the tumor site. The authors expect this novel targeted drug delivery system to have great potential for applications in targeted cancer treatment.

### 3.4. Combination Therapy Involving Cisplatin and Other Drugs

The use of traditional drugs in chemotherapy is associated with many problems such as low water solubility, cytotoxicity, side effects and drug resistance [86,87,88]. A number of potential new drug delivery systems is based on combination therapy that can solve these problems [89,90]. This therapy involves co-administration of drugs that work by different mechanisms against cancer cells.

**Figure 30:**
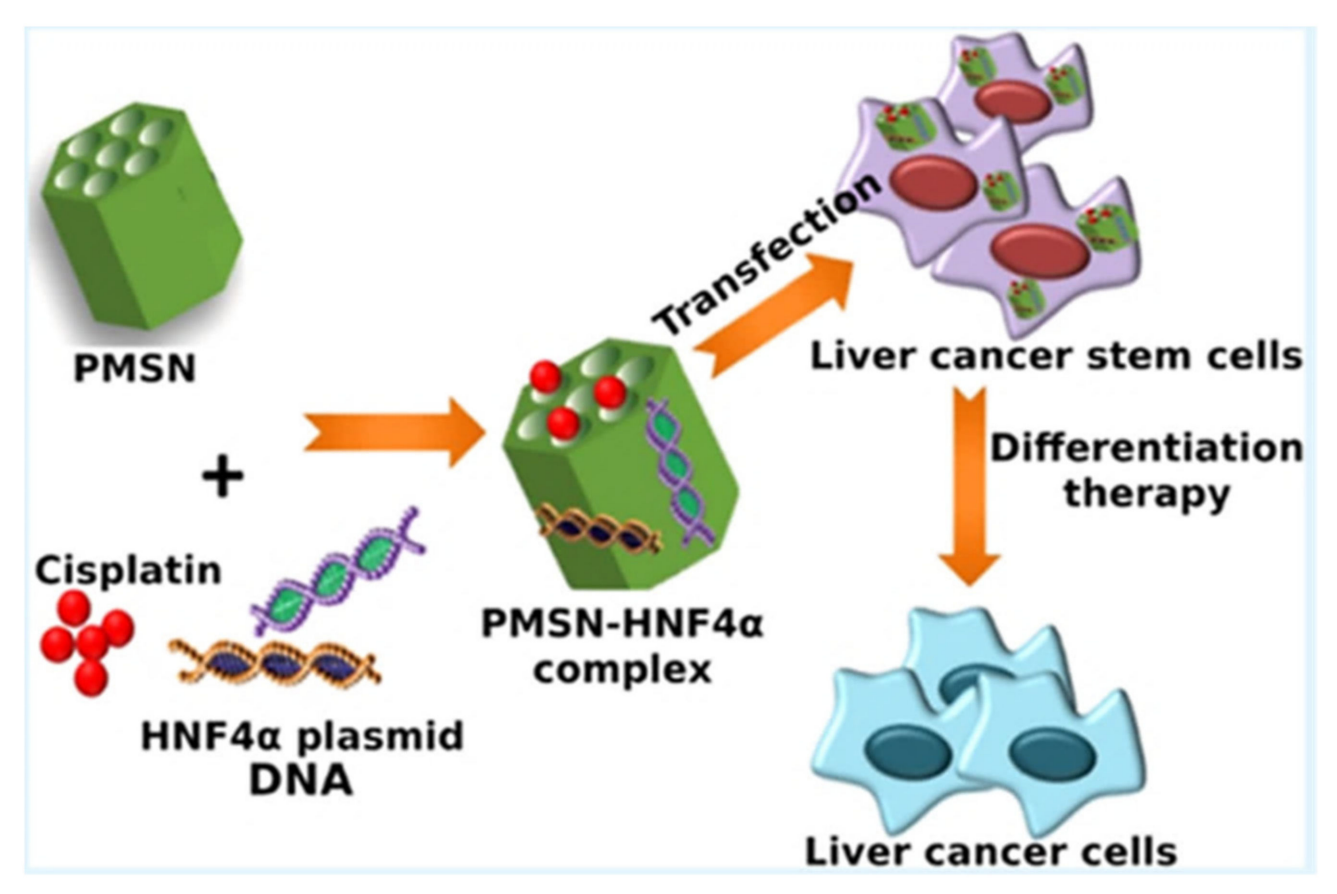
Dual delivery of HNF4α and cisplatin by mesoporous silica nanoparticles [91].

An important approach in cancer therapy is not only the fight against the cancer itself, but also the relapse of the disease caused by the presence of drug-resistant cancer stem cells. A novel approach to the problem has been made via the use of polyethyleneimine-modified mesoporous silica nanoparticles for the co-delivery of cisplatin and a gene encoding HNF4α plasmid (Figure 30) [91]. Such a system is effective in inhibiting the proliferation of Huh7 cell line, reducing the percentage of cancer stem cells, blocking them in the S phase of the cell cycle and leading to apoptosis. The use of the plasmid is crucial because it is a transcription factor responsible for maintaining the heterogeneous state and functional activity of hepatocytes. Negatively charged surface of MSN (110 nm) has been modified with PEI to increase the cell membrane permeability.

**Figure 31:**
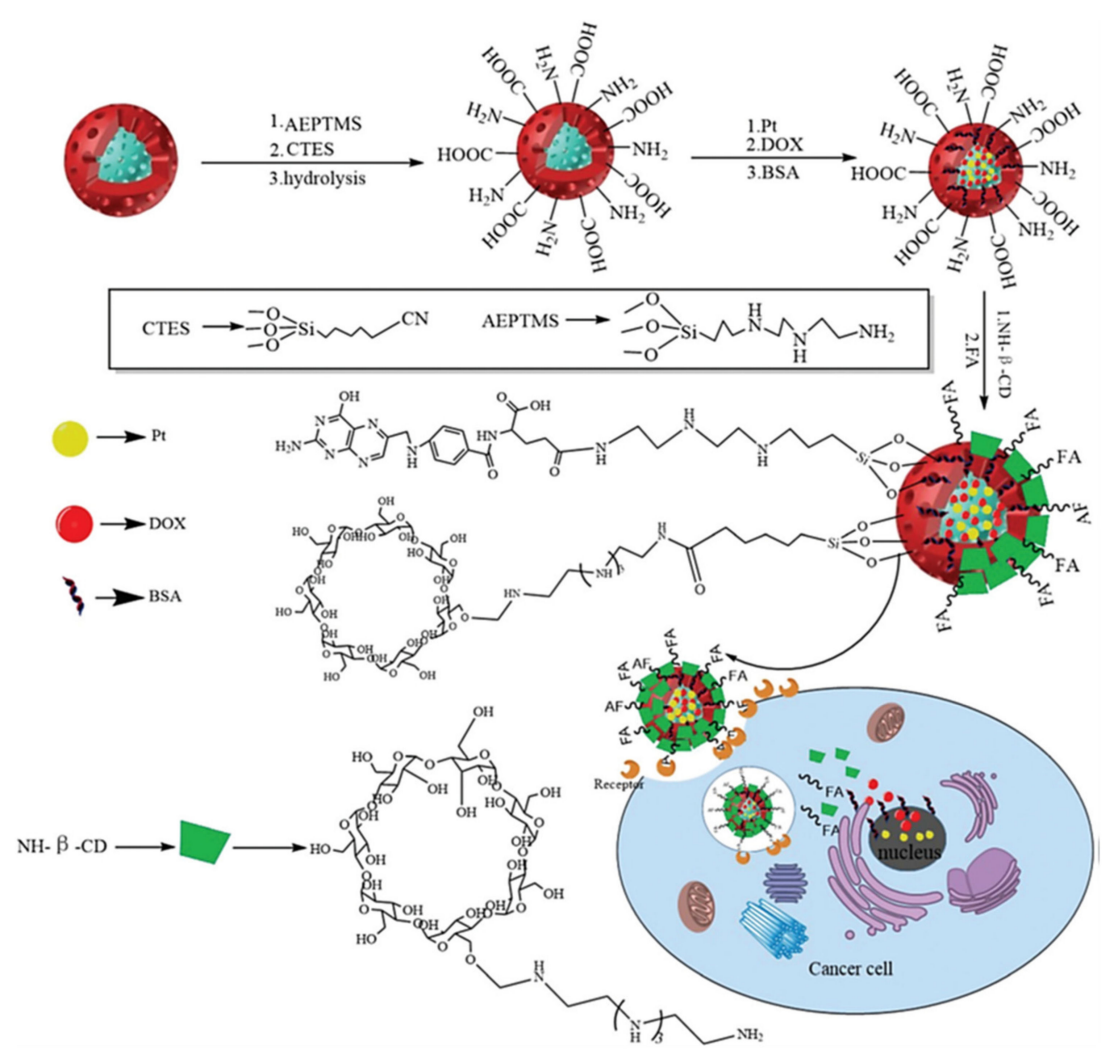
Synthetic scheme for multifunctional double mesoporous silica and schematic loading and release of the drugs and protein [92].

An interesting and complex strategy was also developed in the use of mesoporous silica for co-delivery of cis-platin, doxorubicin and macromolecular model drug—bovine serum albumin (BSA) loaded onto the outer layer using aminoguanidine-cyclodextrin as a blocking agent [92] (Figure 31). The synthesized carrier was made in such the way that its pores could contain both drugs and high molecular weight protein [93]. In order to prevent leakage of cisplatin and doxorubicin, MSN (170 nm) with pore sizes ranging from 2–4 nm and 4–16 nm were synthesized. A very high degree of drug loading was thus achieved. MSN were synthesized using two different cationic surfactants (cetyltrimethylammonium bromide—CTAB and cethylpyridine bromide—CPB). These carriers was simultaneously modified with amino and carboxyl groups and then loaded with drugs. Cyclodextrin and folic acid were covalently attached to the carrier surface to encapsulate drugs and proteins and assure effective targeting. In this way, a system of multi-drug and protein therapy combined with targeted therapy and a controlled release system under the influence of lowered pH was constructed. Although, it has to be stressed that the viability tests against HeLa cells have shown the highest cytotoxicity both for free Pt and free DOX when compared MSN complexes.

An important issue related to the administration of cisplatin is also the resistance of cancer cells associated with the expression of HIF-1 (hypoxia-inducible factor-1) [94]. Antineoplastic drugs activate HIF-1 depending on reactive oxygen species (ROS), then this factor increases the expression of glutathione, which can inactivate cisplatin [95]. Therefore, in cispatin therapy, it is important to inhibit the action of HIF-1 through the action of an inhibitor, e.g., acriflavine. Zhang et al. developed a system in which the cisplatin core was coated with microporous silica in the reverse microemulsion method, and then tetrasulfide-bridged organic silica was incorporated (redox sensitive, degraded by reaction with glutathione) [96]. The nanoparticles were subsequently functionalized with a polymeric mPEG-silane, and finally the cationic acriflavine particles were electrostatically adsorbed on the negatively charged microporous silica coating. Acriflavine in this system successfully prevented the formation of HIF-1α/β dimers, which improved the effectiveness of cisplatin as revealed in in vitro and in vivo studies.

### 3.5. Intelligent Silica Nanoparticles for Cisplatin Delivery

**Figure 32:**
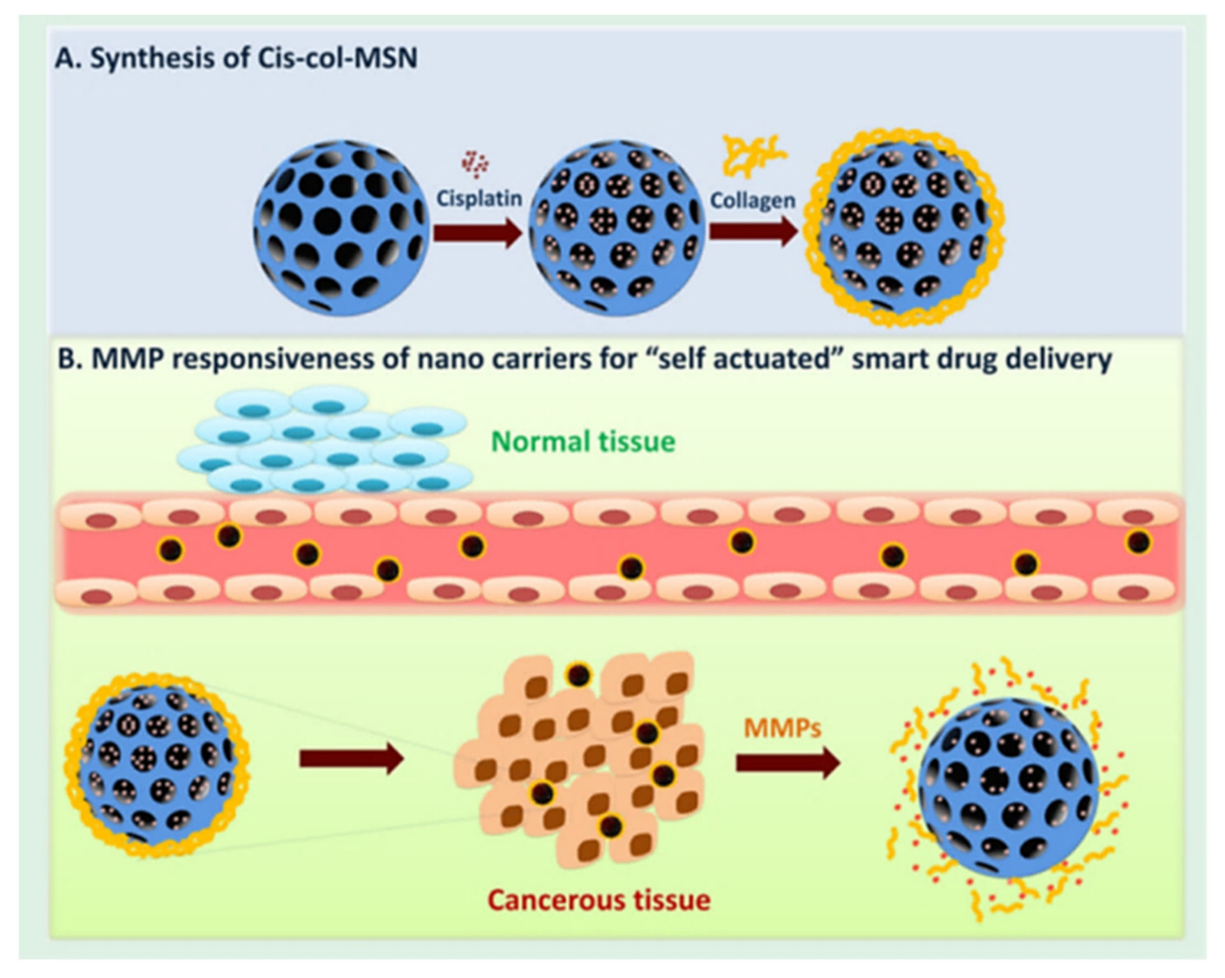
Synthesis of cisplatin-collagen “intelligent” MSN and its action towards normal and cancer cells (A549) [97].

The possibility of modifying the surface of MSN makes it an excellent carrier that releases drugs depending on the stimulus—enzymes, pH, redox, temperature or light. It offers drug release ‘on demand’ and thus treatment control and decrease of the undesirable effects of standard treatment. An intelligent system that provides a controlled release of cis-platin in response to matrix metalloproteinase (MMP) enzymes that are overexpressed in cancer tissues and associated with exacerbation of lung cancer and inflammation [97]. In the proposed system, MSN were loaded with cisplatin and their surface was covered with collagen. Under normal conditions, collagen prevented the release of the drug from MSN, while in the tumor environment, where the MMP enzymes are overexpressed, collagen detaches from the MSN surface and cisplatin is released (Figure 32). The appropriate synthetic approach involved synthesis of MSN by the sol-gel method in an alkaline medium with the participation of the cationic surfactant—CTAB and the silica precursor—TEOS. Thereafter, an amino group was introduced to the surface of the MSN by reaction with (3-aminopropyl)triethoxysilane (APTES) and finally an aldehyde function was formed by treatment of MSN with glutaraldehyde. Such the formed support was loaded with cisplatin and then treated with an aqueous collagen solution which forms (Schiff’s base) bonds between collagen amino groups and aldehyde ones of the support. This intelligent system (190 nm) showed higher in vitro cytotoxicity, cell cycle arrest and apoptosis of lung cancer cells.

**Figure 33:**
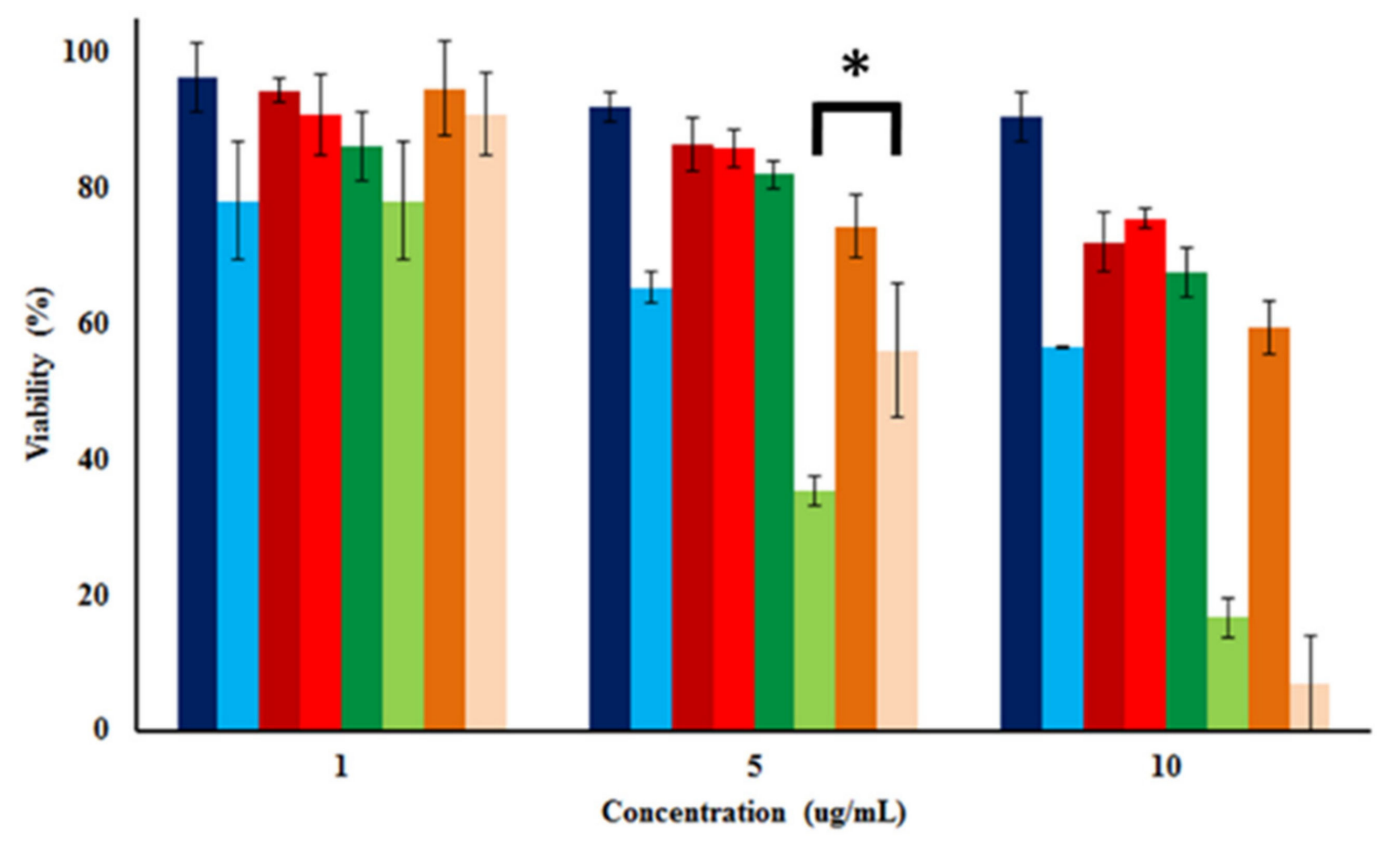
Cytotoxicity towards HeLa cell line of AlClPc-MSNs (dark blue), cisplatin-MSNs (dark red), AlClPc/cisplatin-MSNs (dark green) and physical mixture of cisplatin/AlClPc molecules (dark orange) under dark conditions. Phototoxicity of AlClPc-MSNs (light blue), cisplatin-MSNs (light red), AlClPc/cisplatin-MSNs (light green) and physical mixture of cisplatin/AlClPc molecules (light orange) after red light exposure (570–690 nm; 89 mW/cm^2^) for 20 min [99]. Asterisk indicates *p* < 0.05.

**Figure 34:**
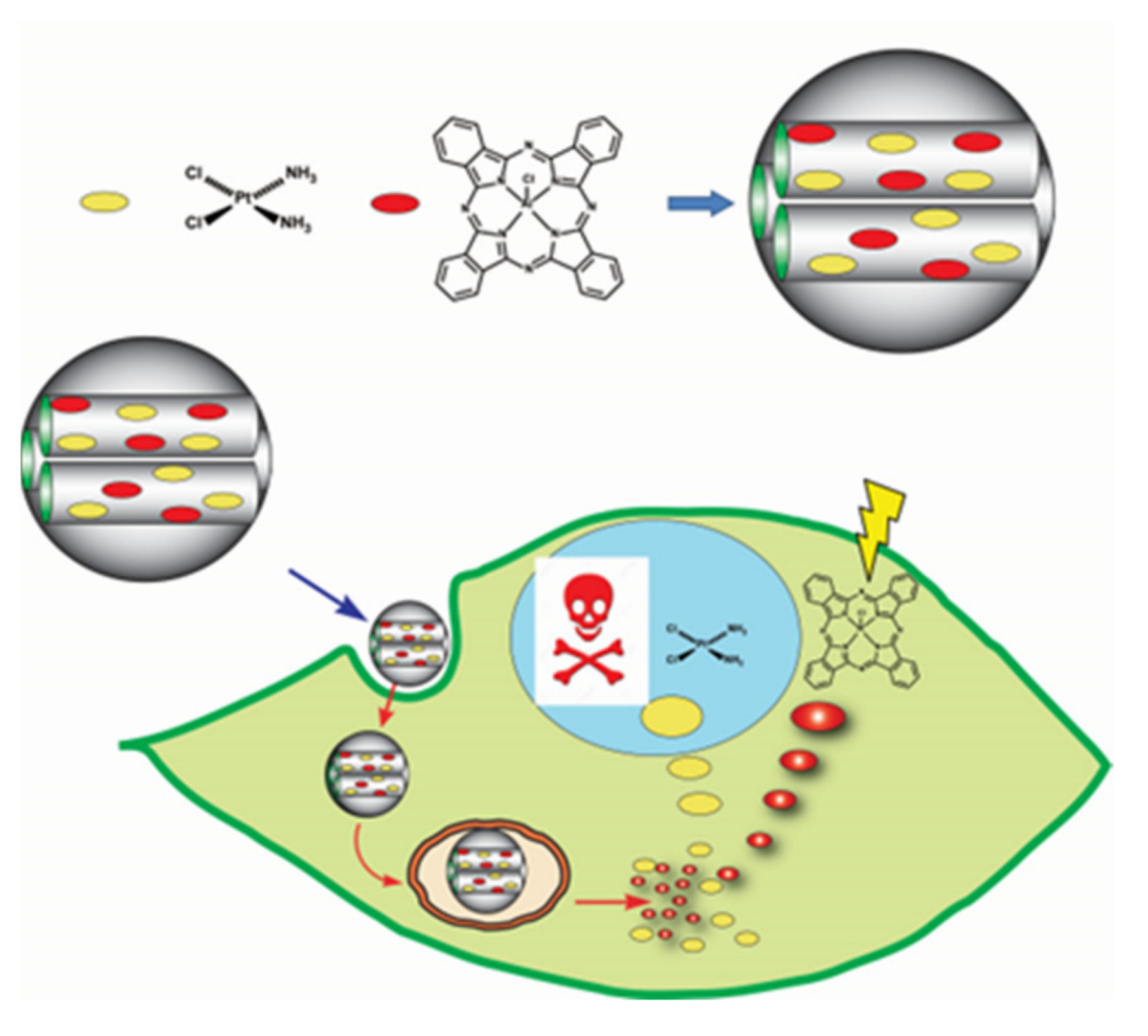
Preparation and action scheme of the system designed for combination chemotherapy and photodynamic therapy in cancer treatment [99].

Mesoporous silica nanoparticles can also be used in photodynamic therapy in conjunction with chemotherapy. The use of a photosensitizer, such as aluminum phthalocyanine chloride (AlClPc), can generate reactive oxygen species toxic to cancer cells only when exposed to light of a specific wavelength [98]. Vivero-Escoto, basing on previous reports on combination chemotherapy and photodynamic therapy for cancer treatment, created a system in which the placement of aluminum chloride phthalocyanine and cisplatin in the pores of MSN nanoparticles intensified toxic effect on cervical cancer cells of HeLa, compared to a physical mixture of cisplatin and AlClPc or their single complexes with MSN [99] (Figure 33 and Figure 34). MSN (approx. 80 nm) were synthesized using TEOS and cetyltrimethylammonium bromide (CTAB) as the template.

**Figure 35:**
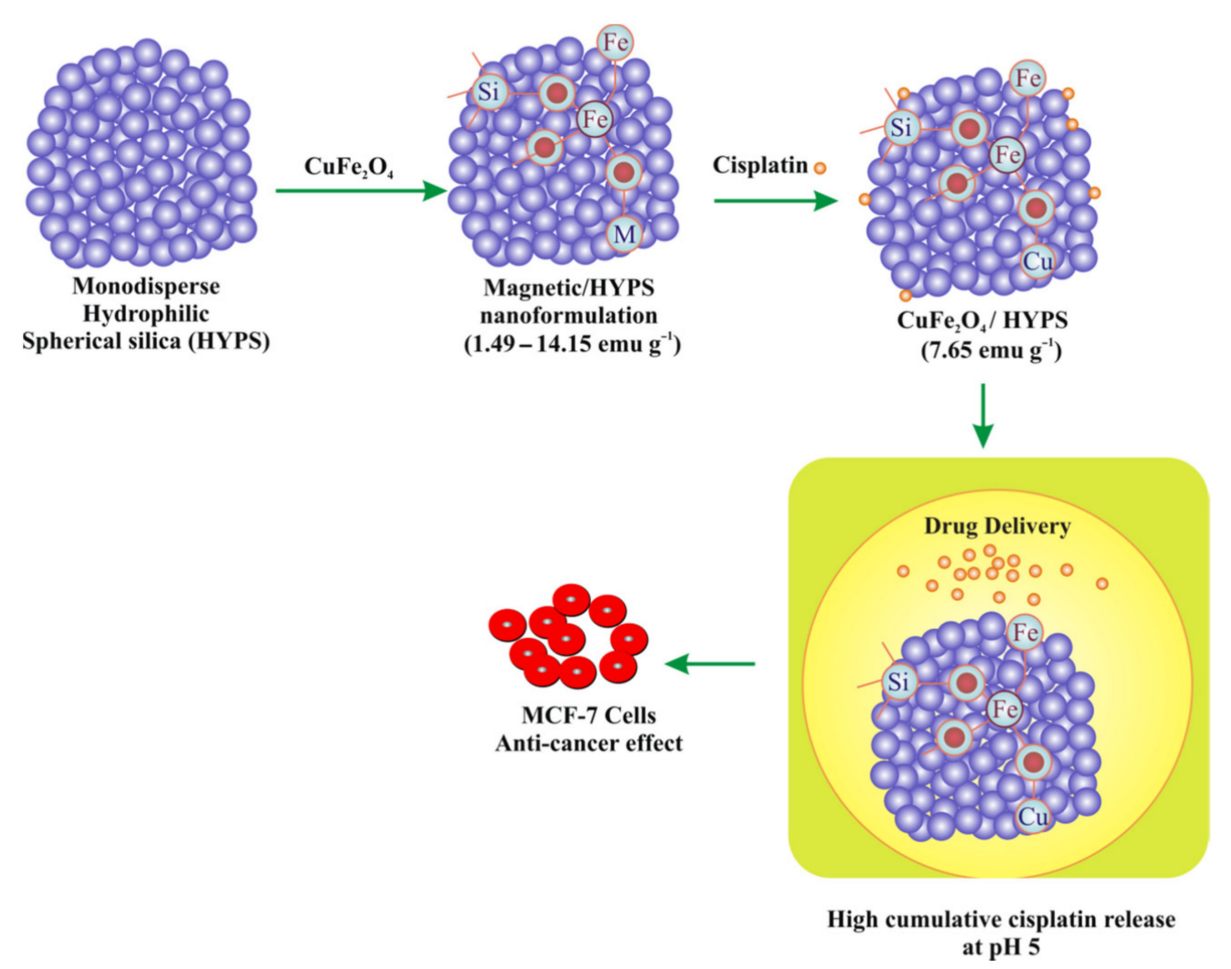
Schematic presentation of CuFe_2_O_4_/HYPS/cisplatin system [100].

An improvement in cis-platin delivery systems can also be achieved by combining magnetic nanoparticles with porous silica. Such systems can respond to an external magnetic field, releasing the drug and assisting with magnetic resonance bioimaging. The use of copper ferrites (CuFe_2_O_4_) is advantageous due to excellent conductive and magnetic properties [100] (Figure 35). Therefore, it was shown [101] that micrometer-sized spherical silica exhibited a very high magnetization of 1.44 emu/g [102] when impregnated 30 wt.% of CuFe_2_O_4_ in the matrix of monodisperse spherical hydrophilic silica (HYPS). The accumulation of copper ferrite nanoparticles on the surface and in the pores of HYPS was confirmed and cisplatin was subsequently loaded by adsorption. The in vitro studies have shown that this system can effectively target MCF-7 breast cancer cells and can be an effective guide for tumor imaging (Table 2).

**Figure 36:**
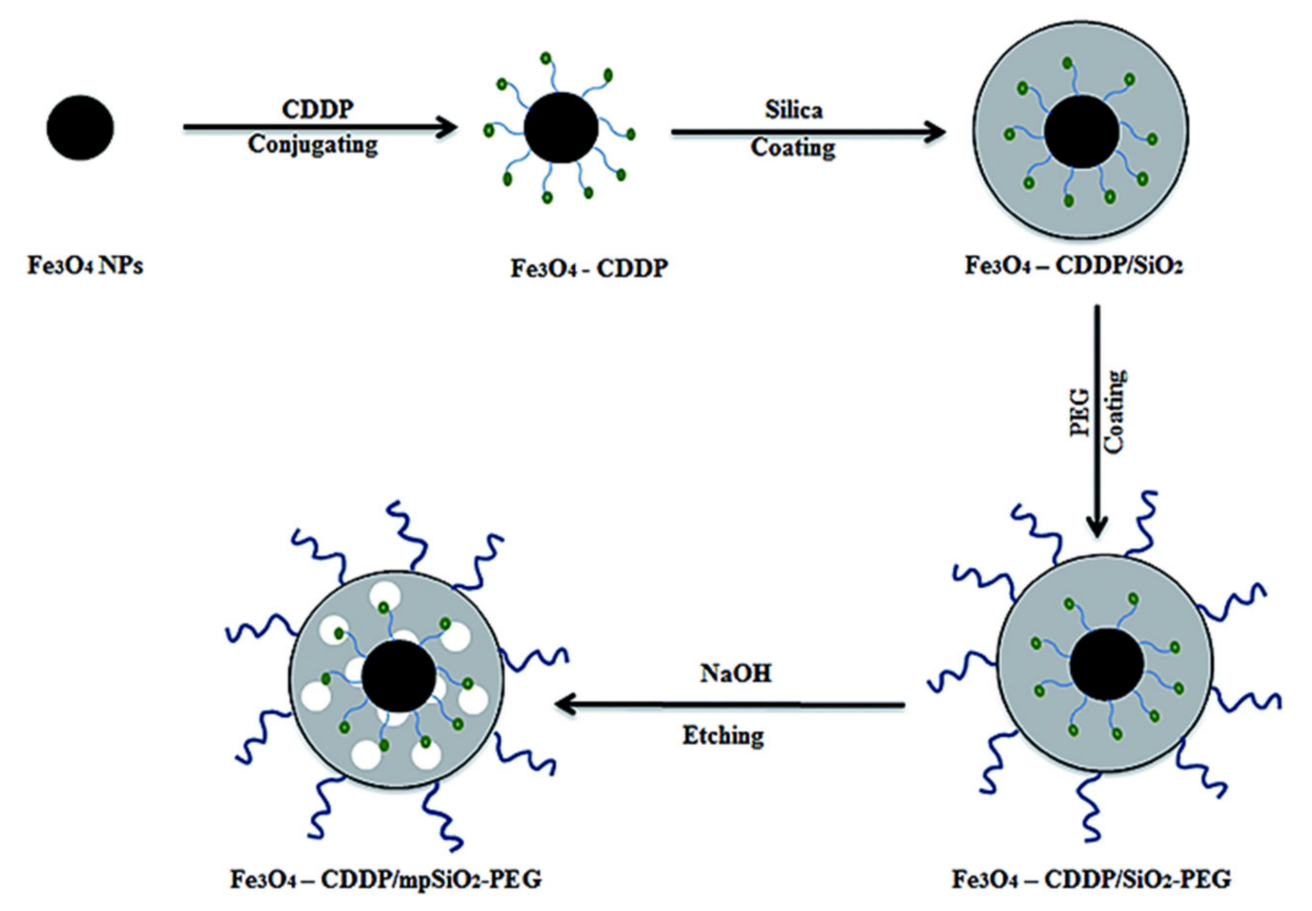
Scheme of the synthesis of the magnetic drug delivery nano-system—Fe_3_O_4_–CDDP [104].

A similar concept of using mesoporous silica nanocarriers equipped with magnetic particles for cisplatin delivery was described by Chandrababu Rejeeth et al. [103]. The core was composed of magnetic particles (Fe_3_O_4_) covalently coupled with cisplatin that was later coated with a layer of PEG-functionalized mesoporous silica (Figure 36). Fe_3_O_4_ nanoparticles were functionalized with methyl 3-mercaptopropionate via the formation of Fe–S bonds. The –OCH_3_ group was then converted into a –NHNH_2_ group by a hydrazinolysis reaction. The process is required as the hydrazide end-groups (–NHNH2) that provide the amide linkage with CDDP are acid-labile with the ability to release the conjugated drug in a weakly acid environment.

A nanocomplex constructed in this way, due to the presence of a mesoporous silica, provided better protection of cis-platin and magnetic nanoparticles, and allowed control of drug release at the target site.

**Figure 37:**
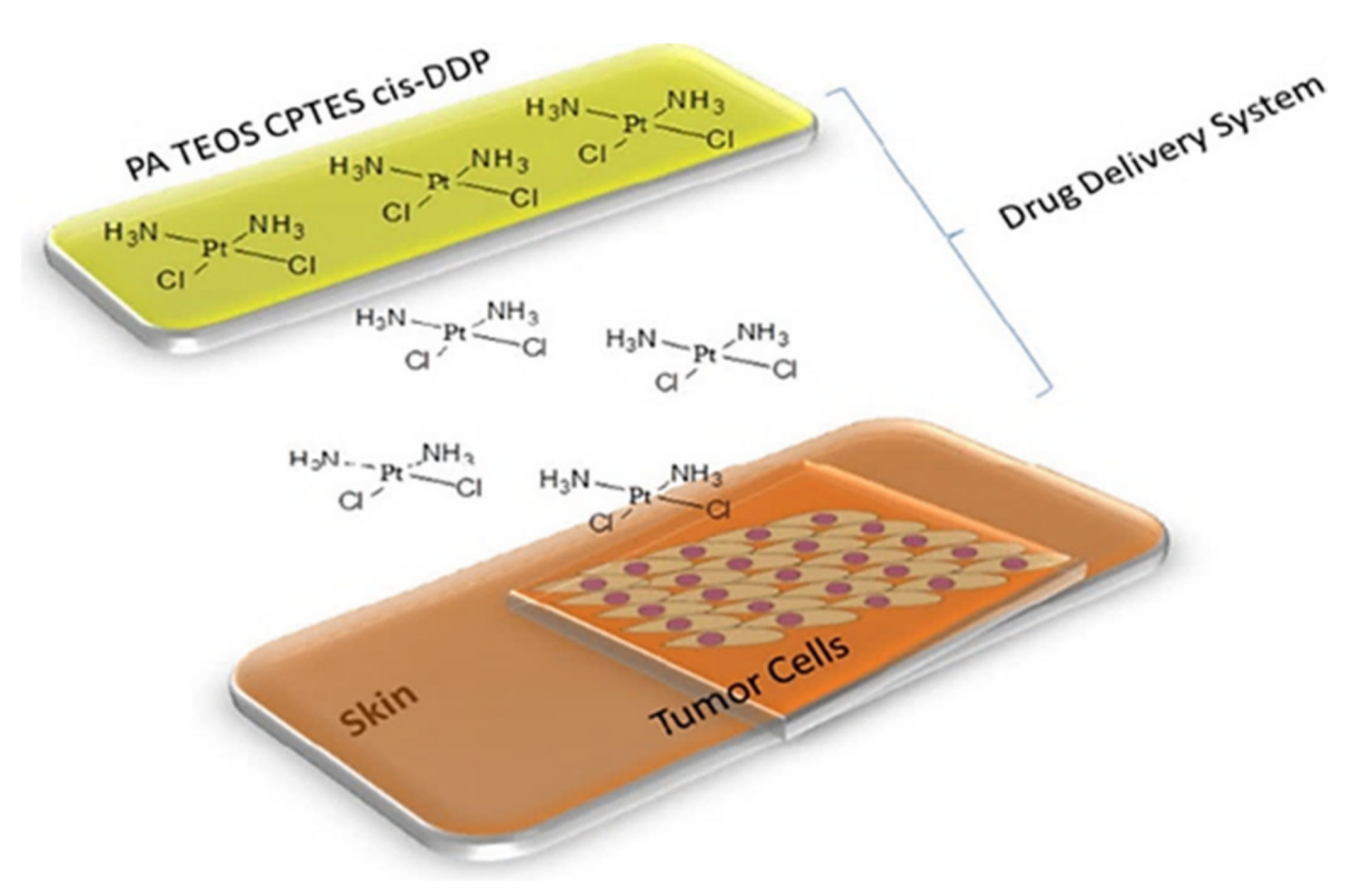

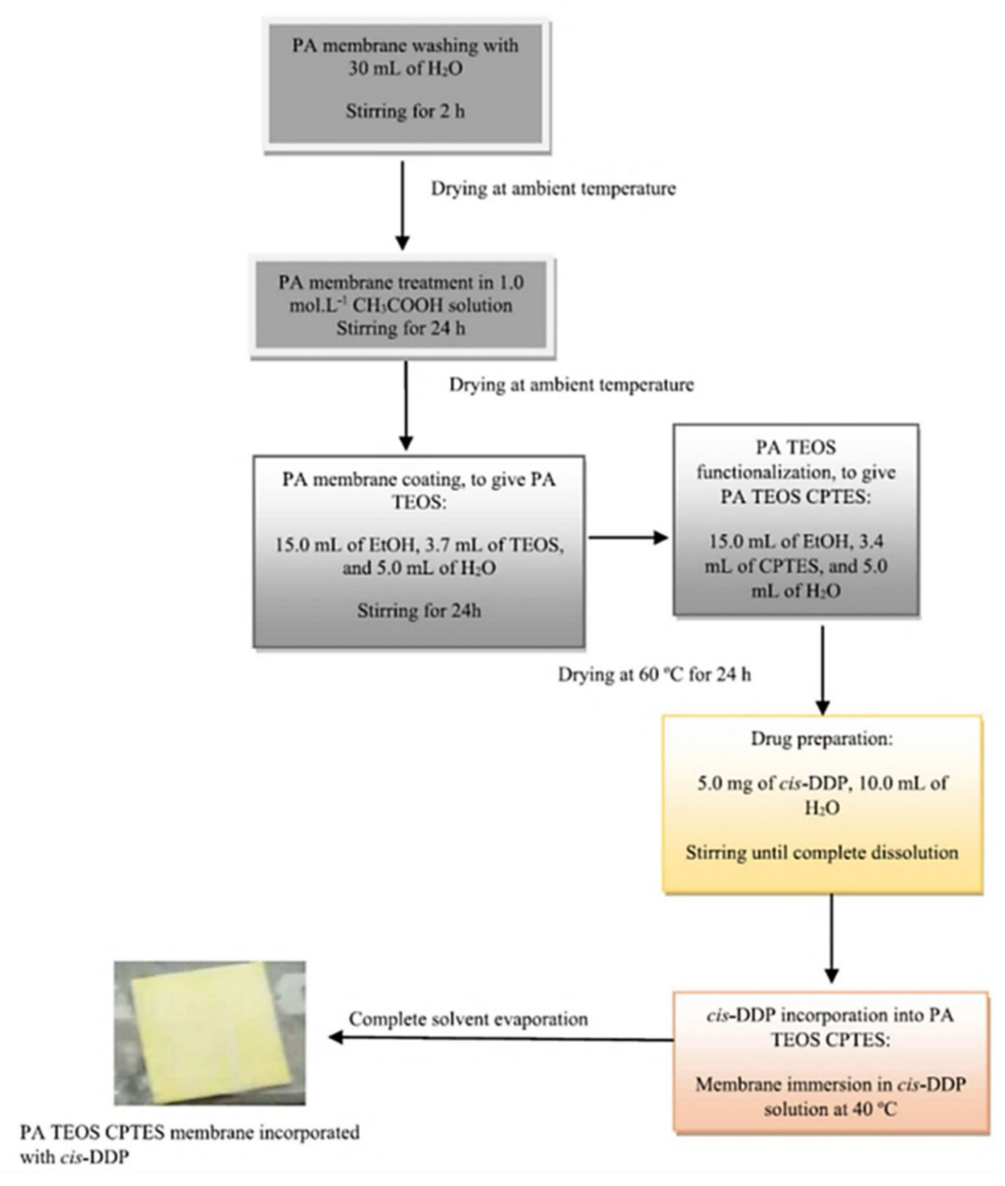
Functionalized polyamide membrane (PA TEOS CPTES) and its preparation [104].

**Figure 38:**
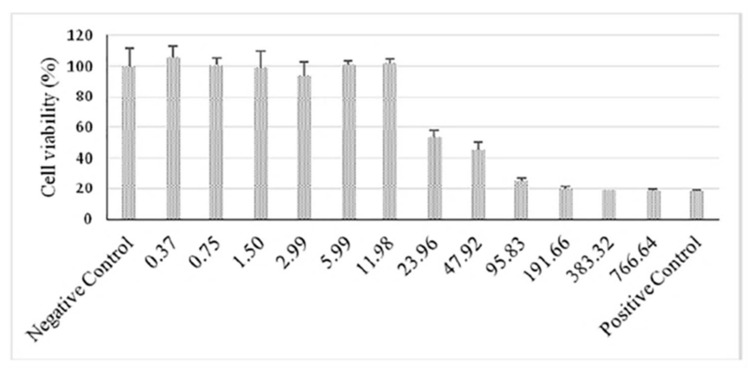
Cell viability assay of GM07492A cell line after treatment with PA TEOS CPTES incorporated with cis-DDP (0.37–766.64 μM) [104].

The gradual release of the drug is also important in the treatment of skin cancer. It was proposed use polyamide membranes activated and functionalized with TEOS and 3-chloropropyltriethoxysilane, followed by incorporation of the antitumor drug—cisplatin [104] (Figure 37). This approach took into account the advantages of using polyamide, i.e., biocompatibility, good mechanical properties and ease of hydrogen bonding with other species [105]. To increase the bioactivity, the surface of the PA membrane was activated with acetic acid and functionalized with silanes. Subsequently cisplatin was introduced by casting at 40 °C from aqueous solution until the solvent was completely evaporated. The studies showed the possibility of incorporating cisplatin into the polyamide membrane. In vitro cytotoxicity studies (XTT assay) against lung human fibroblasts (GM07492A cells) showed that incorporated cis-DDP efficiency remained unaltered after contact with culture medium for 24 h and cis-DPP release therein (IC_50_ = 23.95 μg/M) (Figure 38).

## 4. Conclusions

The nanoarchitectonic approach allowed for the development in recent years of numerous synthetic pathways leading to the preparation molecules and materials for anticancer therapies [106,107,108] and of platinum drugs nano-conjugates and nano-complexes with silicon containing carriers. Physical complexes are easier to be synthesized though the actual covalently bound conjugates often allow the longer release time and smaller administration of doses. In the case of complexes, a similar effect can be achieved by blocking of, e.g., SiO_2_ carrier pores with a stimulus-responsive polymer or the gate-keeping spacious moieties as cyclodextrin. Out of the two the most widely-studied silicon nanocarriers, silsesquioxanes are regarded as the next generation of materials in biomedical fields. They possess organic and inorganic fragments that can be easily functionalized leading to high loading efficiency and thus lower administration dose. The structurally uniform distribution of organic fragments makes silsesquioxanes more effective in terms of activity and hemocompatibility when compared to silicas. A noticeable increase in anticancer effectiveness can be observed once upconversion nanoparticles are introduced into the prodrug delivery systems. Another interesting research direction is the use of magnetic particles (Fe_3_O_4_ or copper ferrites) as components of intelligent nano-carriers of drugs, as well as dual loading of anticancer drugs such as doxorubicin, pemetrexed or nitric oxide in combination with cisplatin. Such materials allow for the combined chemotherapy, as well as photodynamic therapy and imaging. This approach marks a current direction in studies on synthesis and application of novel platinum prodrugs systems involving silicon nanocarriers. The possibility of modifying the surface of silicon based carriers makes them excellent delivery tools that release drugs depending on the stimulus—enzymes, pH, redox, temperature or light. It offers drug release ‘on demand’ and thus treatment control and decrease of the undesirable effects of standard treatment. As mentioned earlier, the silicon based delivery platform offers higher stability, biocompatibility and non-toxicity of such the materials, as well as prolonged release of active platinum drugs. There seems to be a flourishing development of novel platinum drugs delivery systems so clinical and animal tests can be expected in the coming years.

## Figures and Tables

**Table 1:** Summary of studies on organosilicon nanocarrier conjugates with platinum drugs.

Platinum Drug	Organosilicon Nanocarrier	Other Ligand/Carrier/Drug	Targeting Ligand	Cell Line/IC_50_	Size (nm)	Refs.
cisPt(IV)	polysilsesquioxane system	PEG		A549/14.91 μM NCI-H460/2.07 μM	150.3 ± 5	[18]
cisPt(IV)	MNS	mPEG_2k_	lactose (LA)	HepG-2/10.46 μM normal fibroblast L929 cells	-	[22]
cisPt(IV)	MNS	FITC		HeLa/0.22 μM A549/1.07 μM MCF-7/0.38 μM	~2 nm	[38]
cisPt(IV)	MSGNR	β-cyclodextrin, AlPcS_4,_ Ad-PEG, Ad-LA		HepG2 COS7 normal cells	~14 nm (MSGNR)	[42]
cisPt(IV)	UCNPs@SiO_2_	bridge peptide sequence (KKKKKC), oligo(ethyl glycol) (dPEG6)		A2780 A2780cis	-	[44]
hydrated cisplatin prodrugs	MSN	polyacrylamide, doxorubicin		HeLa/0.208 μM(24 h) A357/<0.080 μM(24 h)	291 nm	[46]
cis-diaquadiamino platinum(II)	mesoporous magnetic silica nanoparticles			A2780	225 ± 23 nm	[49]
cis-diaquadiamino platinum(II)	CoFe_2_O_4_ encapsulating silica NPs	rhodamine isothiocyanate (RITC)	folic acid (FA)	HeLa (FR+Ve)/3.0 μg/mL HaCat (FR−ve)/4.6 μg/mL 3T3/7.2 μg/mL	96 nm	[50]
oxaliplatin	MSN-COOH			HepG-2		[51]
cisplatin	Fe_3_O_4_@SiO_2_@Au NPs			SW480 SW620	below 40 nm (Fe_3_O_4_@SiO_2_@Au)	[52]

**Table 2:** Summary of studies on MSN complexes with platinum drugs.

Platinum Drug	Organosilicon Nanocarrier	Other Ligand/Carrier/Drug	Targeting Ligand	Cell line/IC_50_	Size (nm)	Refs.
cisplatin	amine-functionalized mesoporous silica (AMS)	nitric oxide		H596/45.3 μM A549/69.1 μM WI-38/58.6 μM BEAS2B/9.1 μM	~50 nm (AMS)	[64]
cisplatin	MSN-PEG MSN-PEI MSN-SH			-	~140 nm (nanocarriers)	[68]
cisplatin	MSN (containing thiol groups)	6-mercaptopurine		-	98 nm–115 nm	[71]
cisplatin	MSN	PEG-PDS	peptide Arg-Gly-Asp-D -Phe-Cys (cRGDfC)	KB cells	~200 nm (nanocarrier)	[72]
cisplatin	MSN-NH_2_	Polyelectrolyte: cationic poly (allylamine hydrochloride) and negatively charged P(DMA-co-TPAMA), model drug-rhodamine		-	~150 nm (MSN-NH_2_)	[73]
cisplatin	MSNP SiNP			C6	~100 nm	[74]
carboplatin, oxaliplatin, cisplatin	MSN	DOPC lipid ^165^Ho		-	82 nm (^165^Ho-MS)	[63]
activated Pt drugs	MSN	PEG		-	-	[77]
cisplatin	MSN	poly[(N-isopropylacrylamide)-co-(methacrylic acid)]	folic acid	Hep2	-	[82]
cisplatin	MSN		folic acid	LN18/149 μg/mL	100 nm (MNS)	[84]
cisplatin	MSN		vitamin B_12_	-	316 ± 6	[85]
cisplatin	MSN	polyethyleneimine HNF4α		CD133− and CD133+ Huh7 cells	243.1 nm	[91]
cisplatin	MSN	doxorubicin, bovine serum albumin	cyclodextrin and folic acid	HeLa	173 ± 9 nm	[92]
cisplatin	layer of microporous silica and tetrasulfide-bridged organic silica	acriflavine mPEG-silane		A549	-	[96]
cisplatin	MSN functionalized with APTES and glutaraldehyde	collagen		A549	189.6 ± 5.2 nm	[97]
cisplatin	MSN	aluminum chloride phthalocyanine		HeLa/4.2 µM	96.5 ± 10.5 (MNS) 112.7 ± 19.5 (AlClPc/cisplatin-MSNs)	[99]
cisplatin	monodisperse spherical hydrophilic silica (HYPS)	CuFe_2_O_4_		MCF-7	80 nm (HYPS)	[101]
cisplatin	mesoporous silica	magnetic particles (Fe_3_O_4_), PEG		HeLa MCF-7	50 nm	[103]
cisplatin	polyamide membranes			GM07492A/23.95 μgM		[104]

## Data Availability

Not applicable.
